# Comprehensive genomic profiling of high‐grade serous ovarian carcinoma from Chinese patients identifies co‐occurring mutations in the *Ras/Raf* pathway with *TP53*

**DOI:** 10.1002/cam4.2243

**Published:** 2019-05-24

**Authors:** Fangfang Zhong, Tao Zhu, Xuedong Pan, Yanling Zhang, Haikun Yang, Xiangping Wang, Jinwei Hu, Hongxia Han, Lei Mei, Donglin Chen, Kai Wang, Xianrong Zhou, Xiuqin Li, Xiaowei Dong

**Affiliations:** ^1^ Obstetrics & Gynecology Hospital of Fudan University Shanghai China; ^2^ Zhejiang Cancer Hospital Hangzhou China; ^3^ OrigiMed Shanghai China; ^4^ The First Affiliated Hospital of Army Medical University Chongqing China; ^5^ Meizhou People’s Hospital (Huangtang Hospital) Meizhou Hospital Affiliated to Sun Yat‐sen University Meizhou China; ^6^ Nanning Second Peoples Hospital Nanning China; ^7^ Shengjing Hospital of China Medical University Shenyang China

**Keywords:** comprehensive genomic profiling, high‐grade serous ovarian carcinoma, *KRAS*, *TP53*

## Abstract

High‐grade serous ovarian carcinoma (HGSOC) is a major form of ovarian epithelial tumor that is often diagnosed only at an advanced stage when it is already highly aggressive. We performed comprehensive genomic profiling using an analytically validated clinical next‐generation sequencing assay to identify genomic alterations in 450 cancer‐related genes in a cohort of 88 Chinese HGSOC patients. Overall, we detected 547 genomic alterations with an average of 6.2 alterations per tumor. Most of these HGSOC tumors had low tumor mutation burden and were microsatellite stable. Consistent with earlier studies, *TP53* mutations were present in the majority (96.6%) of the tumors studied, and mutations in *BRCA1/2* that affect DNA repair were also detected frequently in 20.5% of the tumors. However, we observed a 10.2% of mutated genes in the *Ras/Raf* pathway, all co‐occurring with *TP53* mutations in the same tumor, which was unrecognized previously. Our results show that in HGSOC patients, there may be an unrecognized co‐occurrence of *TP53* mutations with mutations in *Ras/Raf* pathway.

## 1 INTRODUCTION

Ovarian epithelial tumor is one of the leading causes of cancer‐related deaths in women both in Asia and worldwide.^1^ Understanding the genomic alterations in HGSOC is critical in improving current therapeutic strategies for HGSOC patients. Several studies had been conducted to better understand the molecular characteristics of HGSOC. Analyses by the Cancer Genome Atlas^2^ as well as other groups using smaller cohorts^3^, ^4^, ^5^, ^6^ have repeatedly reported that almost all HGSOC patients harbor mutations in *TP53*, which are presumed to happen during early tumorigenesis^3^ and are a defining feature of HGSOC.^7^ In addition, a smaller proportion of patients also harbor deleterious somatic or germline *BRCA1*/*2* mutations.^2^ On the contrary, low‐grade serous ovarian cancer is characterized by high prevalence of *KRAS* and *BRAF* mutations, and low occurrence of *TP53* mutations.^8^ Thus, *TP53* mutation is a necessary condition for HGSOC, while *KRAS* mutation is generally accepted as a feature of low‐grade serous ovarian cancer.

In this study, we performed comprehensive genomic profiling using an analytically validated clinical next‐generation sequencing (NGS) assay to identify genomic alterations in 450 cancer‐related genes in a cohort of 88 Chinese high‐grade serous ovarian carcinomas, with the aim to better understand the genomic alterations in Chinese HGSOC patients and identify potential opportunities for precision therapy. We show that most of the Chinese HGSOC cases in this cohort also had *TP53* mutations, as reported elsewhere.^9^ To our surprise, we detected that 9 of the 88 (10.2%) Chinese HGSOC tumors had co‐occurring mutations in both *TP53* and genes in the *Ras/Raf* pathway, which was not recognized previously. Preliminary results showed that the co‐occurrence of *TP53* and *KRAS* may more likely to happen in HGSOC patients with endometrial cyst.

## 2 MATERIALS AND METHODS

### 2.1 Study samples

Clinical formalin‐fixed, paraffin‐embedded (FFPE) tumor tissue and matched normal tissue (68 blood and 20 paracancerous tissue) were collected from 88 Chinese HGSOC patients. Of the 88 cases, 39 were randomly extracted from the database of the Department of Pathology, Obstetrics & Gynecology Hospital of Fudan University between 2017 and 2018. Of the 88 cases, 18 were collected from Shengjing Hospital of China Medical University, Shenyang, China. These samples were hospital‐based HGSOC patients enrolled during June 2018 to August 2018. The other 31 samples were randomly collected from 22 hospitals in China from 2017 to 2018. All selected cases were informed, and a written informed consent of the patient was received according to the protocols and procedures approved by the Institutional Review Board. All cases were reviewed and confirmed by at least two independent senior pathologists according to the newest edition of WHO classification.^10^ Immunohistochemistry analysis of p53 protein was performed in all cases. DNA was extracted, and ultra‐deep NGS was performed on hybridization‐captured libraries of 450 cancer genes in a College of American Pathologists (CAP)‐certified laboratory to detect all classes of somatic genomic alterations including substitutions, short and long indels, copy number alterations, and gene rearrangements.

### 2.2 Next‐generation sequencing

Genomic profiling was performed in the laboratory of OrigiMed (Shanghai, China) using the Yuan Su 450 assay. At least 50 ng of cancer tissue DNA was extracted from each 40‐mm^3^ FFPE tumor sample using a DNA Extraction Kit (QIAamp DNA FFPE Tissue Kit) according to manufacturer's protocols. All coding exons of 450 key cancer‐related genes and selected introns of 39 genes commonly rearranged in solid tumors were captured by a custom hybridization capture panel. In addition, the probe density was increased to ensure high efficiency of capture in regions with low read depth. Libraries were each diluted to 1.05 nmol L^−1^ and then sequenced with a mean coverage of 900× for FFPE samples and 300× for matched blood or paracancerous samples on an Illumina NextSeq‐500 Platform.

### 2.3 Bioinformatics analysis

Reads were aligned to human reference genome hg19 using BWA.^11^ Single nucleotide variants (SNVs) and short indels were called by MuTect^12^ following deduplication, base quality recalibration, and local realignment using GATK^13^ and in‐house pipeline. Short indels were further calibrated using Pindel.^14^ Copy number variations (CNVs) were called using customized algorithms from the log‐ratio per gene region after normalizing read depths within target regions by EXCAVATOR.^15^ A customized algorithm was used to estimate tumor cellularity based on allele frequencies of the sequenced single‐nucleotide polymorphisms (SNPs), and detect gene rearrangements, fusions, and long indels. Reliable somatic alterations were detected by comparison with matched normal samples. At least 5 reads were required to support alternative calling. For CNVs, focal CNVs were characterized as genes with ≥ 5 copies for amplification and 0 copies for homozygous deletion. Clinically relevant genomic alterations were further marked as druggable genomic alterations if they match current treatments or clinical trials.

Tumor mutation burden (TMB) was estimated by counting somatic mutations including coding base substitutions and indels per megabase (Mb) of the sequence examined. Known cancer driver mutations and germline alterations in dbSNP were excluded from the TMB calculation. MSI status was inferred based on MANTIS^16^ score, and microsatellite regions were manually reviewed in Integrated Genomics Viewer (IGV)^17^ for confirmation.

## 3 RESULTS

### 3.1 Patient demographics

Overall, 88 Chinese HGSOC patients were included in this study (Table 1). The morphological features of all cases conformed to typical morphology of HGSOC, composed of solid masses of cells with slit‐like spaces. Papillary, glandular, or cribriform areas can also be seen in some cases. The tumor cells were severely atypical and had an aberrant p53 phenotype, as demonstrated by either diffuse, strong immunostaining or totally negative for p53. Of the 88 cases, 4 were with endometriotic cysts in the same or the opposite ovary. The median age at the time of sequencing was 53 years (range 29‐82 years). Out of 88 samples, 14 harbor germline *BRCA1*/*BRCA2* mutations. The *BRCA* germline mutation carriers were significantly younger than the non‐carriers (*t* test *P* value = 1.8e‐7), indicating strong genetic predisposition. Most patients were at stage III (n = 36, 40.9%) or IV (n = 31, 35.2%) of the disease. Of the 88 (86.4%) samples, 76 were from the original primary tumor, and 11 (12.5%) were from metastatic tumor sites including liver (n = 4, 4.5%), abdominal wall (n = 3, 3.4%), lymph node (n = 2, 2.3%), pelvic wall (n = 1, 1.1%), and intestine (n = 1, 1.1%). The samples taken from tumor metastatic sites were taken at the time of recurrence. Out of 88 patients, 14 received neoadjuvant chemotherapy before surgery.

**Table 1:** Patient demographics

Item	Description
Number of patients	88
Age, median (range)	53 (range 29‐82)
Stage	I: 5 (5.7%)
	II: 8 (9.1%)
	III: 36 (40.9%)
	IV: 31 (35.2%)
	Unknown: 8 (9.1%)
Site	Primary: 76 (86.4%)
	Metastatic: 11 (12.5%), including liver (4), abdominal wall (3), lymph node (2), pelvic wall (1), and intestine (1)
	Unknown: 1 (1.1%)
Tumor mutation burden, average (range)	5.2/Mb (0.8‐39/Mb)
Microsatellite status	MSI‐high: 3
	MSS: 85
Family history	Of 48 patients with data available, 14 (29.2%) have known family history of cancer
Genomic alterations, total and average (range)	547, average 6.2 per sample (range 1‐15)
Actionable genomic alterations	56 (10.2% of all mutations), in 40 tumors (45.4% of all tumors)

### 3.2 Genomic alterations identified in the Chinese HGSOC cohort

**Figure 1:**
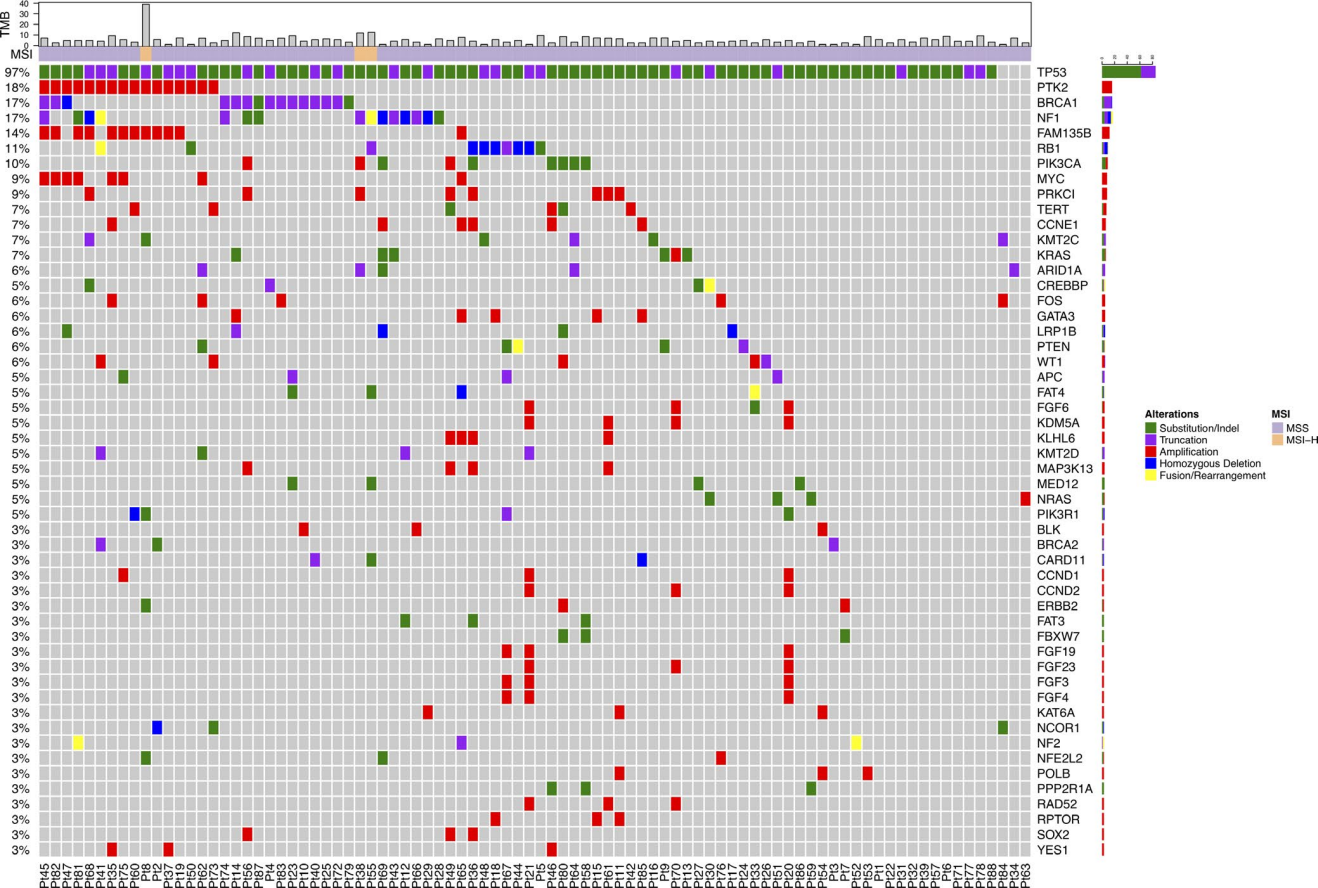
Tile plot of the genomic alterations identified in three or more tumors in this Chinese HGSOC cohort

A total of 547 genomic alterations (including 529 somatic alterations and 18 germline alterations of *BRCA1/2*) were detected in these 88 HGSOC tumors, with an average of 6.2 alterations per tumor (range 1‐15; Figure 1). The most frequently mutated gene in this cohort was *TP53*, which was mutated in 85 of the 88 (96.7%) samples. *BRCA1*/*2* were mutated in 18 of the 88 (20.5%) samples, and more *BRCA1* (n = 16 mutations in 15 [17.0%] patients) than *BRCA2* (n = 3, 3.4%) mutations were observed. One patient (Pt14) harbored both somatic and germline *BRCA1* mutations. Most *BRCA1/2* mutations were truncations, and 14 of the 19 (73.7%) *BRCA1*/*2* mutations were identified from the germline samples. Amplifications in *PTK2*, which encodes focal adhesion kinase (FAK), also had high prevalence in this cohort, as it was detected in 16 of the 88 (18.2%) samples. Other frequently mutated genes in this cohort included *NF1* (n = 15, 17.0%), *FAM135B* (n = 12, 13.6%),* RB1* (n = 10, 11.4%), *PIK3CA* (n = 9, 10.2%), *MYC* (n = 8, 9.1%), *PRKCI* (n = 8, 9.1%), and *KRAS* (n = 6, 6.8%), *TERT* (n = 6, 6.8%), *CCNE1* (n = 6, 6.8%), *KMT2C* (n = 6, 6.8%), and *PTEN* (n = 5, 5.7%). This was followed by a long tail of other genes that were altered less frequently in the cohort. The full details of mutations occurred in three or more patients in this cohort were shown in Figure 1.

Tumors in this cohort had an average tumor mutation burden (TMB) of 5.2 mutations per megabase (Mb; range 0‐39 mutations/Mb; see Methods). Most tumors had low tumor mutation burden, and only 4 of the 88 tumors (4.5%) had more than 10 mutations/Mb. Of the 88 (3.4%) tumors, all with TMB higher than 10 mutations/Mb, 3 were tested to be microsatellite instability‐high (MSI‐H), and the remaining 85 tumors (96.6%) were microsatellite stable (MSS; see Methods). Details of the TMB and microsatellite status for each tumor were shown in Figure 1.

### 3.3 Co‐occurrence of point mutations in the *Ras/Raf* pathway

**Figure 2:**
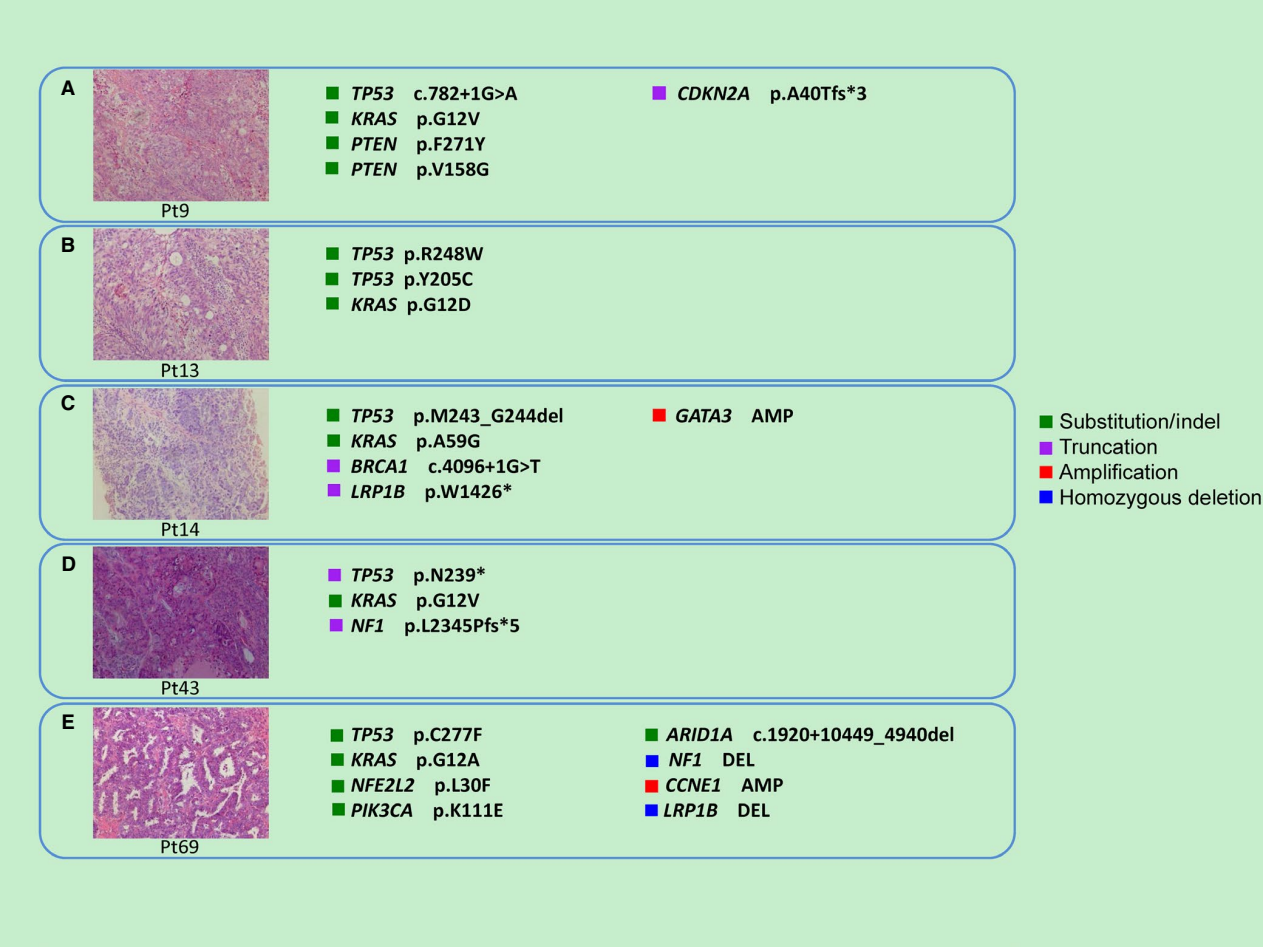
Hemotoxylin and eosin staining of HGSOC tumors in this cohort with co‐occurring *KRAS* point mutation and *TP53* mutations. Total magnification = 10 × 20 = 200×. (A) Pt9, (B) Pt13, (C) Pt14, (D) Pt43, and (E) Pt69. (A‐D) The nuclei of high‐grade serous carcinoma are larger with greater pleomorphism and large nucleoli, diffuse, solid, or nested pattern. (E) Endometrioid carcinoma‐like pattern, irregular serrated luminal contours, and high‐grade nuclei

Point mutations in *KRAS* were identified in 5 of the 88 (5.7%) tumors in this cohort, including 4 hotspot *KRAS* mutations at amino acid 12 (2 G12V, 1 G12A, and 1 G12D), and 1 less common *KRAS* A59G mutation. Notably, all of these cases had co‐occurring *TP53* mutations in the same tumor (Table 2). Pathological review confirmed that all five cases were HGSOC (Figure 2). In addition to *KRAS*, other notable point mutations in the *Ras/Raf* pathway in this cohort included three *NRAS* mutations (1 Q61R, 1 Q61K, and 1 G12C) and one *BRAF* mutation (D594N). All these tumors also had a co‐occurring *TP53* mutation (Table 2). Combined, 9 of the 88 tumors (10.2%) in this Chinese HGSOC cohort had point mutations in the *Ras/Raf* pathway that co‐occur with *TP53* mutations. All these nine co‐occurrences were validated by Sanger sequencing or ddPCR (Supplementary Material Chromas.pdf and ddPCR.pdf). This is the first time that the co‐occurrence of mutated *TP53* and *KRAS* was reported in HGSOC patients.

**Table 2:** Co‐occurring point mutations in genes in the *Ras*/*Raf* pathway with *TP53* mutations

Individual ID	*TP53* mutation	VAF (*TP53*)	*KRAS* mutation	VAF (*KRAS*)	*NRAS* mutation	VAF (*NRAS*)	*BRAF* mutation	VAF (*BRAF*)
Pt9	c.782 + 1G > A	0.44	p.G12V	0.50				
Pt13	p.R248W	0.06	p.G12D	0.01				
	p.Y205C	0.01						
Pt14	p.M243_G244del	0.43	p.A59G	0.17				
Pt43	P.N239*	0.26	p.G12V	0.42				
Pt69	p.C277F	0.57	p.G12A	0.34				
Pt30	p.P64Afs*85	0.41			p.Q61R	0.42		
Pt51	p.R342*	0.15			p.G12C	0.12		
Pt59	p.R273H	0.87			p.Q61K	0.41		
Pt36	c.920‐2A > G	0.67					p.D594N	0.37

### 3.4 Comprehensive genomic profiling revealed many clinically relevant mutations

Of the 547 (10.2%) genomic alterations in this cohort, 56 were associated with targeted therapies that could potentially lead to clinical benefits for the patients. Notable alterations included those occurring in genes involved in DNA repair genes *BRCA1/2* or *ATM* (20 mutations in 19 tumors (21.6%) combined), which sensitize the tumor for poly (ADP‐ribose) polymerase (PARP) inhibitors.^18^, ^19^ Of these, somatic *BRCA1* mutations were identified in 4 (4.5%) tumors, including 2 truncating mutations (E699* and C1382Sfs*11), 1 homozygous deletion, and 1 single nucleotide variant (SNV) that affected a splice donor site (c.4096 + 1G > T). Germline *BRCA1* mutations were identified in 12 (13.6%) tumors, including 10 truncating mutations (5 nonsense mutations including Q126*, F901*, Q1200*, Q1299*, and Q1458*; 5 frameshift deletions including S281Afs*17, Y655Vfs*18, S873Ffs*30, I1824Dfs*3, and E1257Gfs*9) and 2 missense SNVs (R1495K and W1837C). One of the tumors (Pt14) harbored both a somatic *BRCA1* mutation (c.4096 + 1G>T) and a germline *BRCA1* mutation (S281Afs*17). *BRCA2* mutations were identified in 3 (3.4%) tumors (all in germline samples), including 2 truncating mutations (both frameshift deletions, A938Pfs*21 and I2675Nfs*5) and 1 large in‐frame deletion of exons 1‐11. Somatic *ATM* truncating mutation was identified in 1 tumor (K1057*).

## 4 DISCUSSION

Comprehensive genomic profiling has become standard practice in cancer cares in recent years with the advancement of NGS technologies and their reduced costs. Here, we studied the mutational profiles of 88 Chinese HGSOC tumors using comprehensive genomic profiling of 450 cancer‐related genes, and were able to reveal a wide range of 547 mutations in all categories (base substitutions, short insertions and deletions, gene rearrangements and fusions, and copy number changes), many of which were clinically relevant. Our results confirmed previous reports about several frequently mutated genes in HGSOC including *TP53* and *BRCA1/2*. Moreover, we also found that mutations in a few important cancer genes, including genes in the *Ras/Raf* pathway (*KRAS*, *NRAS*, and *BRAF*).

*TP53* was mutated in 96.6% tumors in this cohort, which is consistent with many earlier sequencing studies on HGSOC that established *TP53* as the dominant mutation in this cancer.^2^, ^3^ Previously, the presence of mutations in *TP53* had been proposed as a defining feature of HGSOC.^7^ The three tumors in our cohort which were *TP53* mutation negative could possibly be explained by *TP53* mutations outside the coding region with unknown function, or *TP53* mutations at low frequency below our detection threshold. Hemotoxylin and eosin staining of HGSOC tumors from these three individuals were attached in Supplementary Figures. Although no targeted therapies are yet available for these *TP53* mutations, in a recent study, tumor infiltrating T‐cell responses to two *TP53* hotspot mutations G245S and Y220C were identified in the context of HLA‐DRB3*02:02 in two separate ovarian cancer patients.^20^ Among the tumors sequenced in this cohort, five harbored the *TP53* Y220C mutation and one had the G245S mutation. Although HLA typing was not performed for this cohort, HLA‐DRB3*02:02 had over 22% population frequency among the Chinese population in the US.^21^ This suggests that adoptive T‐cell therapy could be a promising direction for some Chinese HGSOC patients, and further studies are needed to better understand their efficacy and identify additional immunogenic *TP53* hotspot mutations and relevant HLA‐restriction types.

We observed five co‐occurrences of mutated *TP53* and *KRAS* in our cohort. We then hypothesize that the co‐occurrence was correlated with endometrial cyst, which was more likely to evolve to clear cell carcinoma or endometrioid carcinoma. If this is the case, then the co‐occurrence of mutated *TP53* and *KRAS* could possibly be explained by a mixture of endometrial cyst and high‐grade ovarian carcinoma. By revisiting the clinical data, we were able to identify two endometrial cyst cases out of a total of five patients with mutated *TP53* and *KRAS*. At the same time, we observed 2 endometrial cyst cases in the other 83 patients. To test the independence between *TP53* and *KRAS* co‐occurrence and endometrial cyst status, we applied Fisher's exact test on the contingency table (Table S1) and found that *TP53* and *KRAS* co‐occurrence was significantly dependent on endometrial cyst status (*P* value = 0.015). Thus, the co‐occurrence of *TP53* and *KRAS* could partly be explained by a mixture of HGSOC and endometrial cyst.

*BRCA1*/*2* germline and somatic mutations were identified in 18 of the 88 (20.5%) patients in this cohort, also at a frequency similar to what had been reported elsewhere.^2^, ^22^ *BRCA1/2* are key homologous recombination (HR) genes that play crucial roles in DNA double‐strand break repair. Ovarian cancer patients with *BRCA1/2* mutations are known to respond better to platinum‐based chemotherapies and have longer overall survivals^2^, ^23^ and they also benefit from PARP inhibitor which is currently the most important targeted therapy for HGSOC patients.^24^ The high prevalence of *BRCA1/2* mutations, most of which were from germline samples, highlighted the importance of routine *BRCA1/2* testing for Chinese ovarian cancer patients. Several other tumors in the cohort also harbored mutations in other HR genes including *ATM*, *RAC1*, and *RAD51C*.

Mutations in the *Ras/Raf* pathway and in *PIK3CA* showed higher prevalence in this Chinese HGSOC cohort. We found that 9 of the 88 (10.2%) tumors in this cohort had point mutations in *KRAS* (n = 5), *NRAS* (n = 3) or *BRAF* (n = 1) in addition to *TP53* mutations. *KRAS* and *BRAF* had been reported to mutate more frequently in low‐grade serous ovarian carcinoma^8^, ^25^ as well as other histological subtypes of ovarian cancer,^26^ yet their clinical significance in HGSOC is unknown.^26^ Recently, a study reported that *KRAS* mutation in ovarian cancer predicts that the tumor may be sensitive to MEK inhibition.^27^ *PIK3CA* mutations had been reported previously to occur more frequently in endometrioid and clear cell ovarian cancers^28^, ^29^ but rarely in HGSOC.^2^ Further studies are needed to understand the significance of such co‐occurrences of mutations in the *Ras/Raf* pathway or *PIK3CA* (Table 3) with *TP53*.

**Table 3:** Co‐occurring point mutations in *PIK3CA* with *TP53* mutations

Individual ID	*TP53* mutation	VAF (*TP53*)	*PIK3CA* mutation	VAF (*PIK3CA*)
Pt35	R342*	0.72	P539R	0.42
Pt46	R273H	0.71	M1043V	0.44
Pt58	R273H	0.08	C378W	0.07
Pt64	R248W	0.40	E542K	0.26
Pt69	C277F	0.57	K111E	0.49
Pt80	R273H	0.56	K111N	0.34

*PTK2* amplifications were identified in 16 of the 88 (18.1%) tumors in this cohort. Of the 16 tumors with *PTK2* amplifications, 11 (68.8%) also had co‐amplifications in *FAM135B,* a gene located very close to *PTK2* on the chromosome 8*. PTK2* is located on chromosome 8q24.3 which had been linked to ovarian cancer susceptibility.^30^ It encodes focal adhesion kinase (FAK), which is a critical component in transmitting signals from extracellular environments into the cell, and its activation in cancer drives tumor progression and metastasis.^31^ Frequent *PTK2* amplification had been reported in ovarian as well as other cancers,^32^, ^33^, ^34^ and was associated with poor overall survival.^35^ Recently, several FAK inhibitors have been developed and are being tested in clinical trials (NCT01138033, NCT01943292, and NCT00787033). If proved effective, they could bring clinical benefits to these HGSOC patients with *PTK2* amplification.

Two tumors in this cohort (2.3%; Pt10 and Pt18) harbored amplifications in both *CD274* (PD‐L1) and *PDCD1LG2* (PD‐L2). Moreover, 3 of the 88 (3.4%) tumors in this cohort were tested to be MSI‐H (Pt8, Pt38, and Pt55; Figure 1). Together, these 5 (5.7%) tumors might benefit from checkpoint inhibitor treatment. Despite this, several recent early phase clinical trials applying checkpoint inhibitors in ovarian cancer had demonstrated only limited efficacy with response rate between 5% and 20%.^36^ Further identification of useful biomarkers is needed to better stratify patients and identify those that are more likely to respond. In addition, several ongoing clinical trials are studying the effect of combining checkpoint inhibitors with chemotherapies (NCT02520154), targeted therapies (NCT02484404), or other checkpoint inhibitors (NCT02498600) in ovarian cancers, and results from these trials may provide important insights on how to optimally apply immunotherapy for such patients.

In summary, comprehensive genomic profiling has revealed an unrecognized co‐occurrence of *TP53* mutations with mutations in *Ras/Raf* pathway, for example, *KRAS* and *NRAS*, and detected mutations that are informative for choosing personalized treatment regimens in almost half of all the tumors studied in this cohort of Chinese HGSOC patients, demonstrating its values in routine clinical practice.

## CONFLICT OF INTEREST

XP, JH, HH, LM, DC, KW, and XD are employees of OrigiMed.

## AUTHOR CONTRIBUTIONS

FZ, TZ, XL, and XD wrote the paper. XP, XL, and XD conceived this idea. XZ, XL, and XD reviewed the cases. YZ, HY, XW, and HH manipulated the data. JH, LM, and DC annotated the variants.

## Data Availability

The data that support the findings of this study are available from the corresponding author upon reasonable request (dongxw@origimed.com).
