# Supplementary material for: Comprehensive genomic profiling of high‐grade serous ovarian carcinoma from Chinese patients identifies co‐occurring mutations in the Ras/Raf pathway with TP53
Source: Cancer Med. 2019 May 24;8(8):3928–35. doi: 10.1002/cam4.2243 (PMC6639185; doi:10.1002/cam4.2243)

Comprehensive genomic profiling of high-grade serous ovarian carcinoma from Chinese patients identifies co-occurring mutations in the *Ras/Raf* pathway with *TP53*

Supplementary Figures

H&E Staining of HGSOC tumors from the three individuals within whom we were not able to detect *TP53* mutations. (Discussed in the Discussion section.)

Pt33

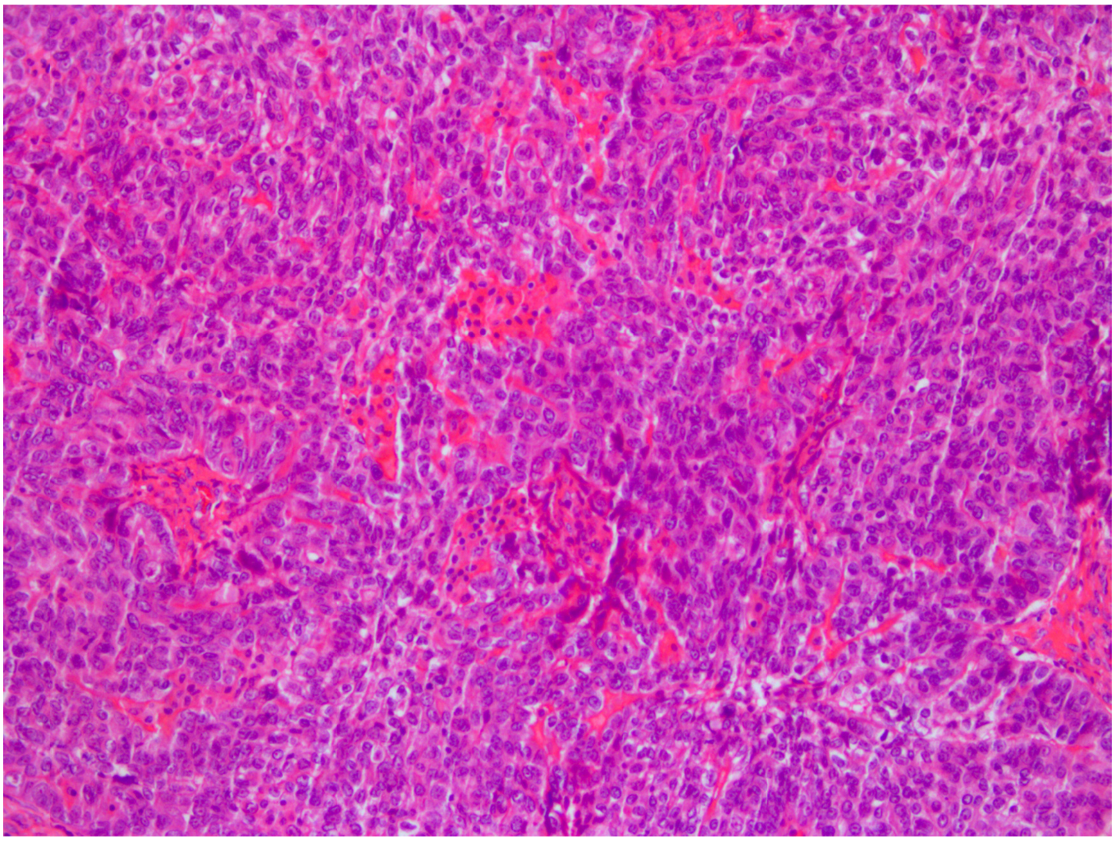

Pt63

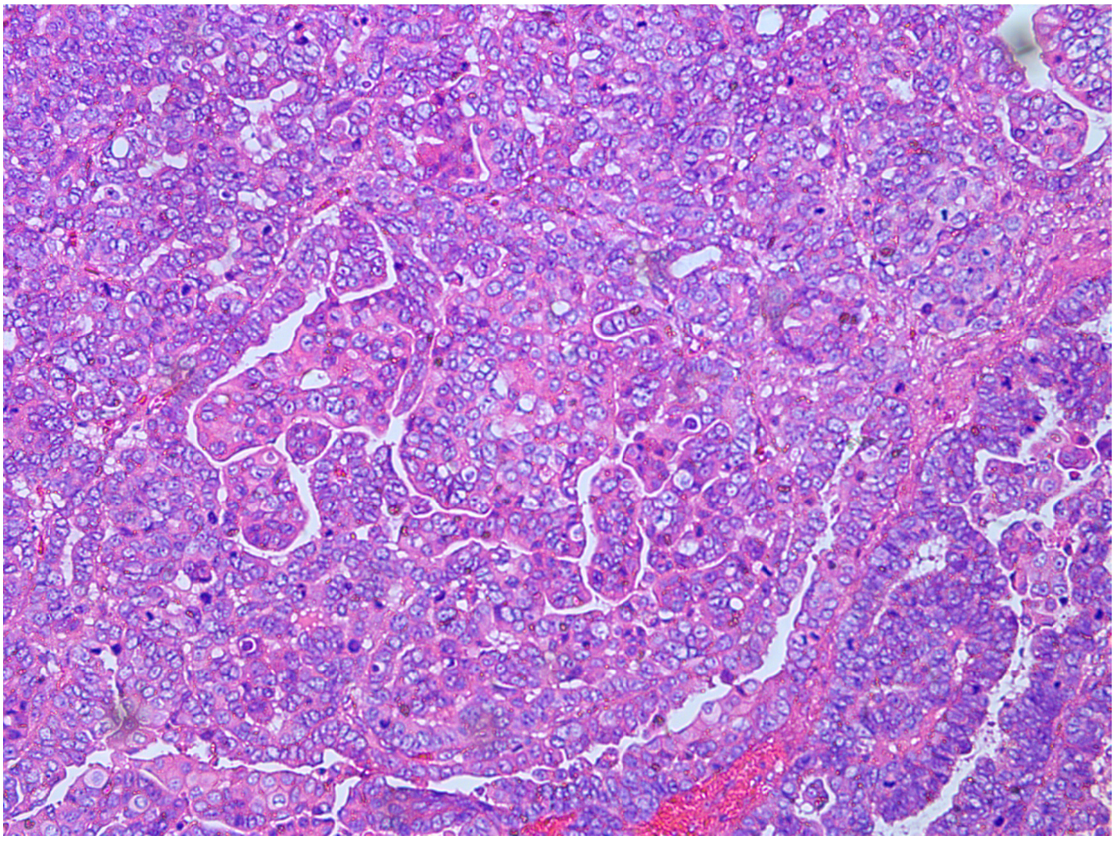

Pt85

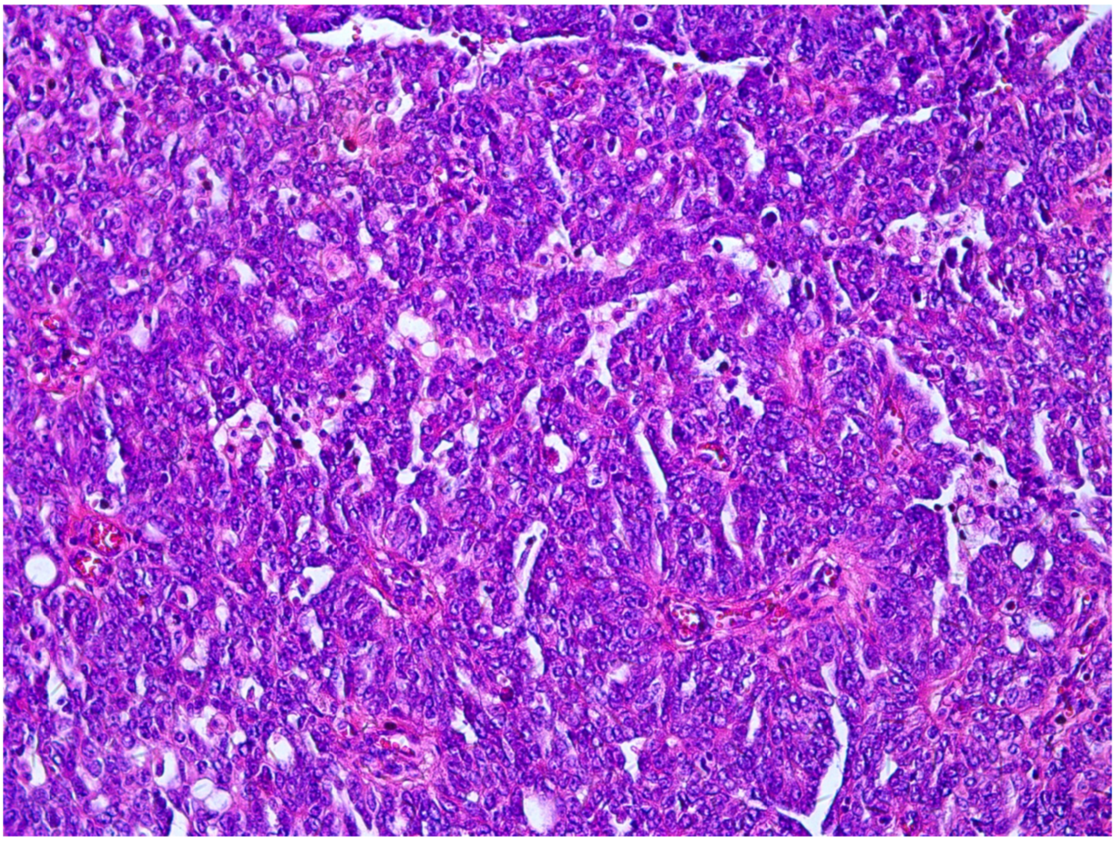

Supplement: Supplementary file 1 [file CAM4-8-3928-s001.pdf]
