# Supplementary material for: Comprehensive genomic profiling of high‐grade serous ovarian carcinoma from Chinese patients identifies co‐occurring mutations in the Ras/Raf pathway with TP53
Source: Cancer Med. 2019 May 24;8(8):3928–35. doi: 10.1002/cam4.2243 (PMC6639185; doi:10.1002/cam4.2243)

**Supplementary Documents**

H&E Staining of HGSOC tumors from the three individuals within whom we were not able to detect *TP53* mutations. (Discussed in the Discussion section.)

**Pt33**


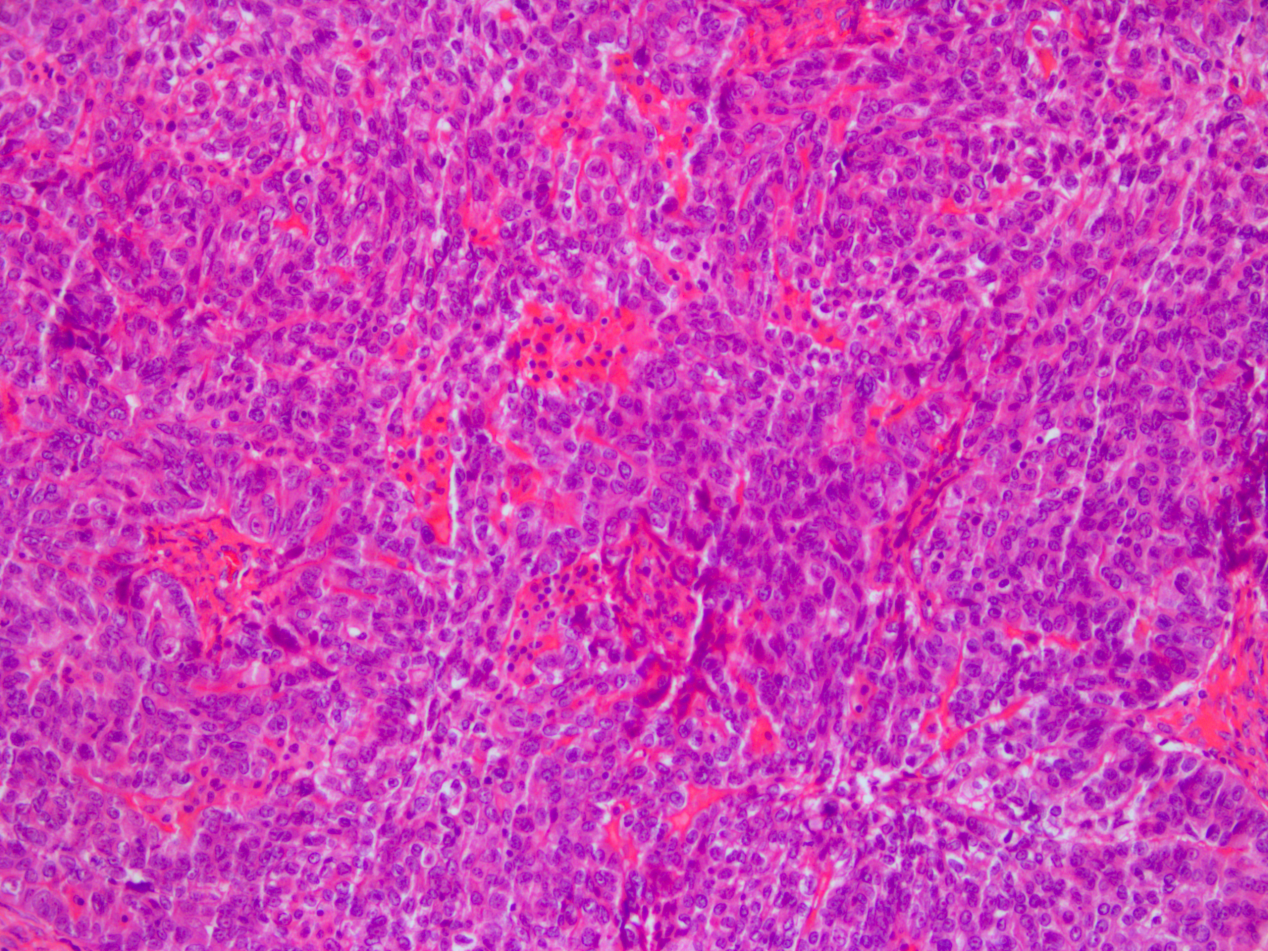


**Pt63**


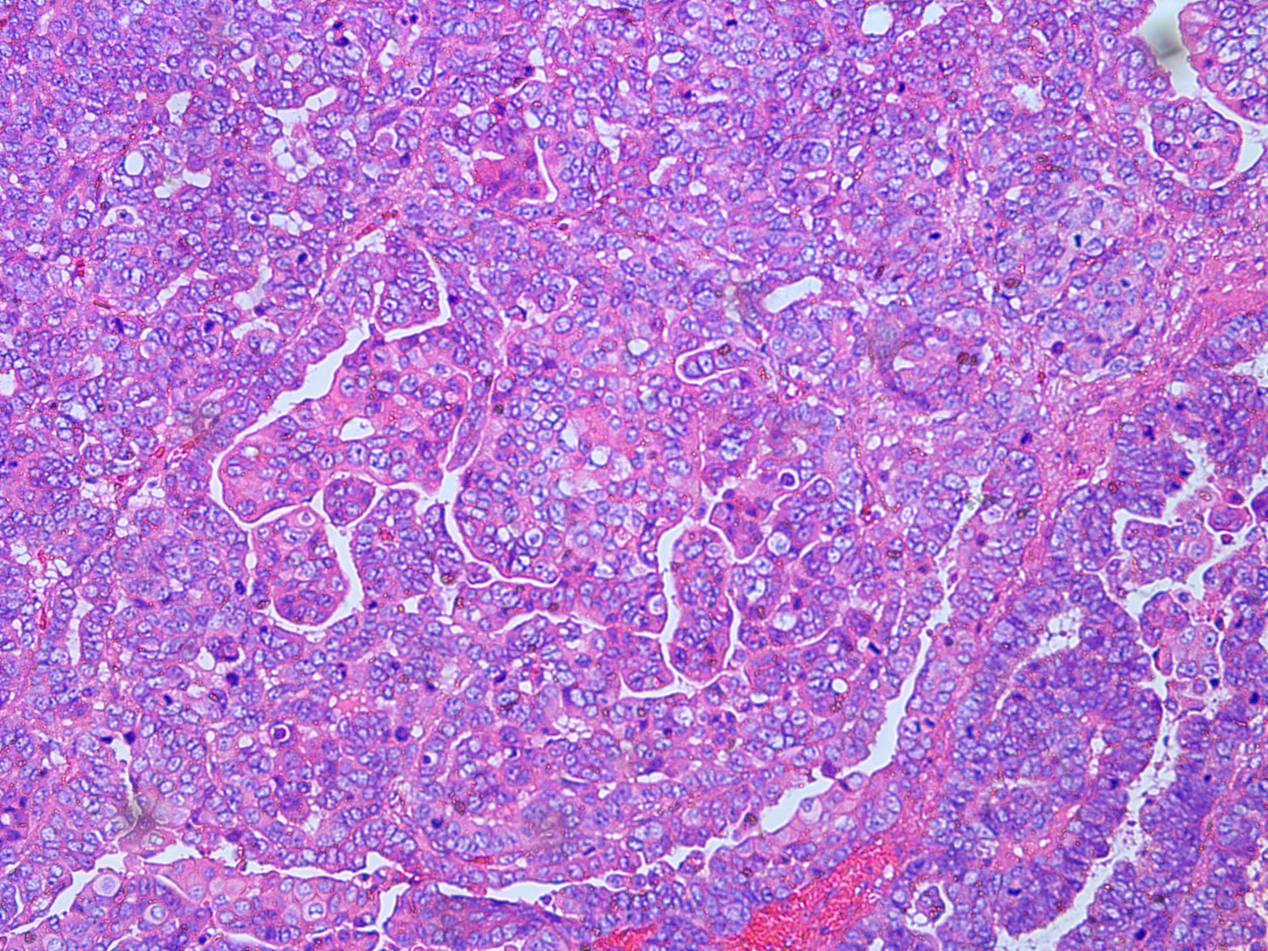


**Pt85**


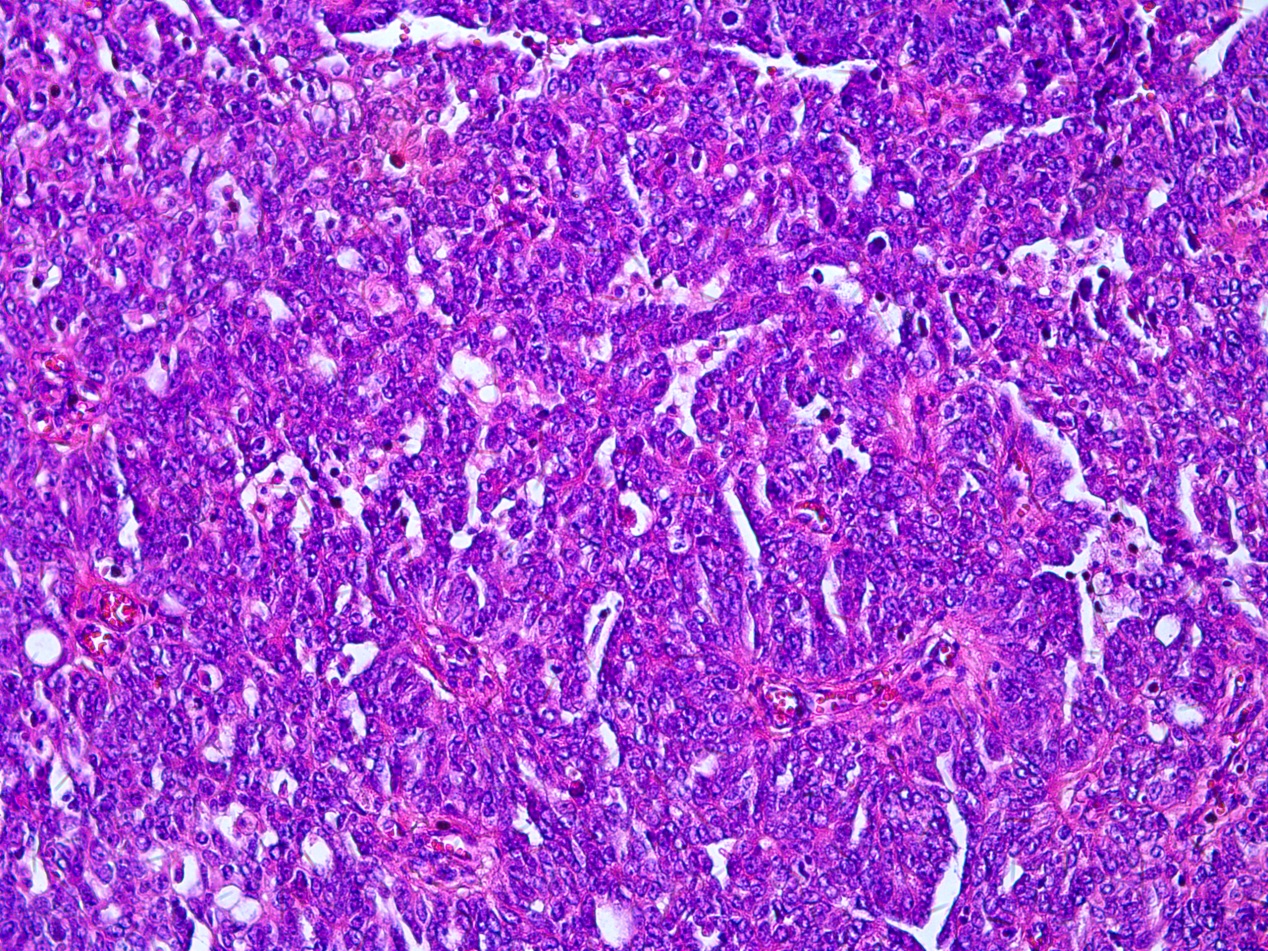

Supplement: Supplementary file 3 [file CAM4-8-3928-s003.docx]
