# Supplementary material for: Comprehensive genomic profiling of high‐grade serous ovarian carcinoma from Chinese patients identifies co‐occurring mutations in the Ras/Raf pathway with TP53
Source: Cancer Med. 2019 May 24;8(8):3928–35. doi: 10.1002/cam4.2243 (PMC6639185; doi:10.1002/cam4.2243)
Supplement: Supplementary file 4 [file CAM4-8-3928-s004.pdf]

Pt13

TP53 NM_000546 c.742C>T p.R248W

NGS VAF = 0.06 ddPCR VAF = 0.0693

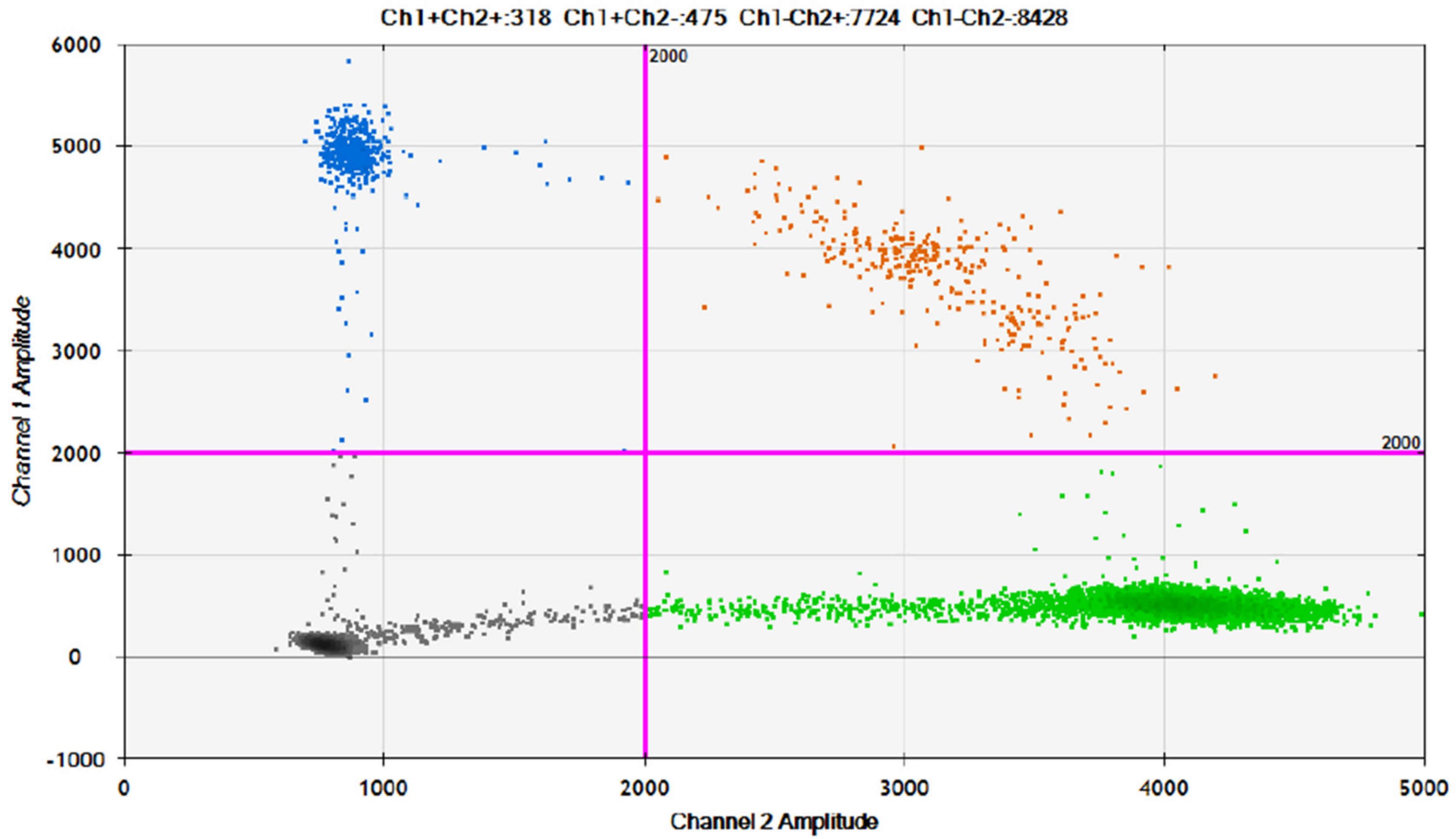

Accepted Droplets	Concentration（copies/ul）	Copies Per 20uL Well	ddPCR VAF	DNA loadded (ng）
16945	56.4	1128	6.93%	30.00
	757	15140		

Pt13

KRAS NM_004985 c.35G>A p.G12D

NGS VAF = 0.01 ddPCR VAF = 0.0067

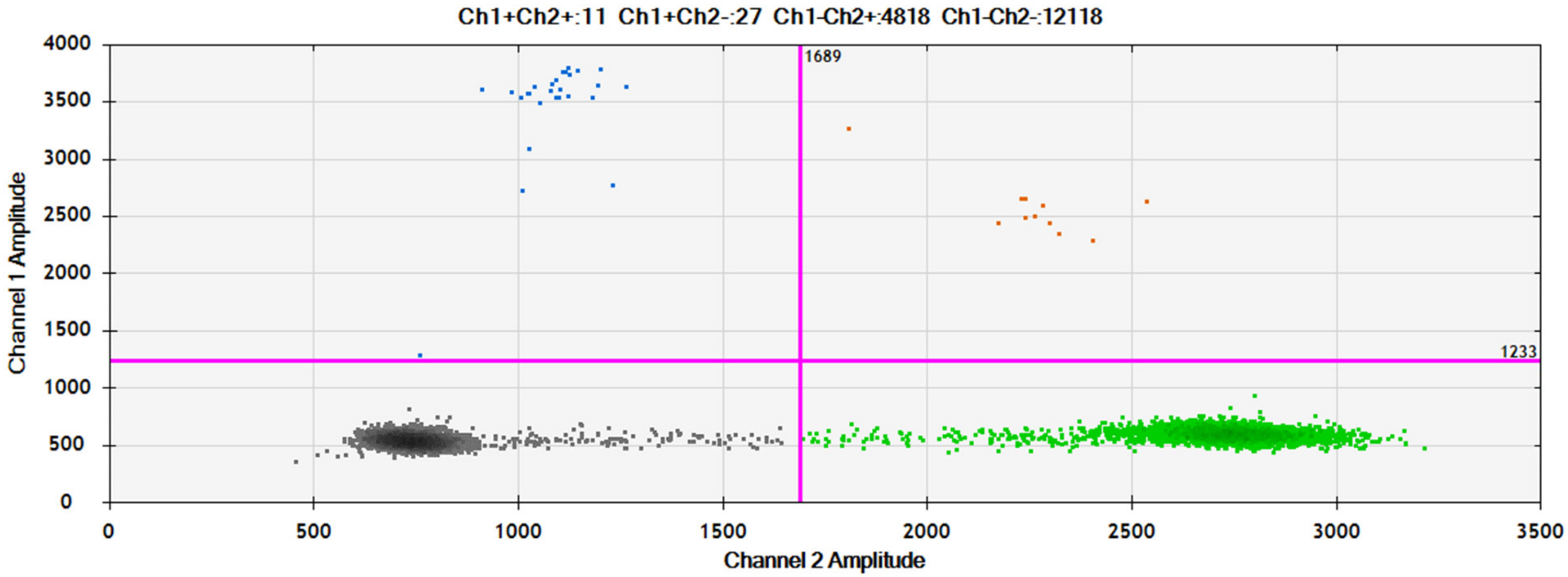

ddPCR mutant （copies/20ul)	ddPCR wildtype （copies/20ul)	ddPCR percentage of mutant	DNA loaded (ng)
52	7880	0.67%	8.00

Pt51

NRAS NM_002524 c.34G>T p.G12C

NGS VAF = 0.12 ddPCR VAF = 0.1292

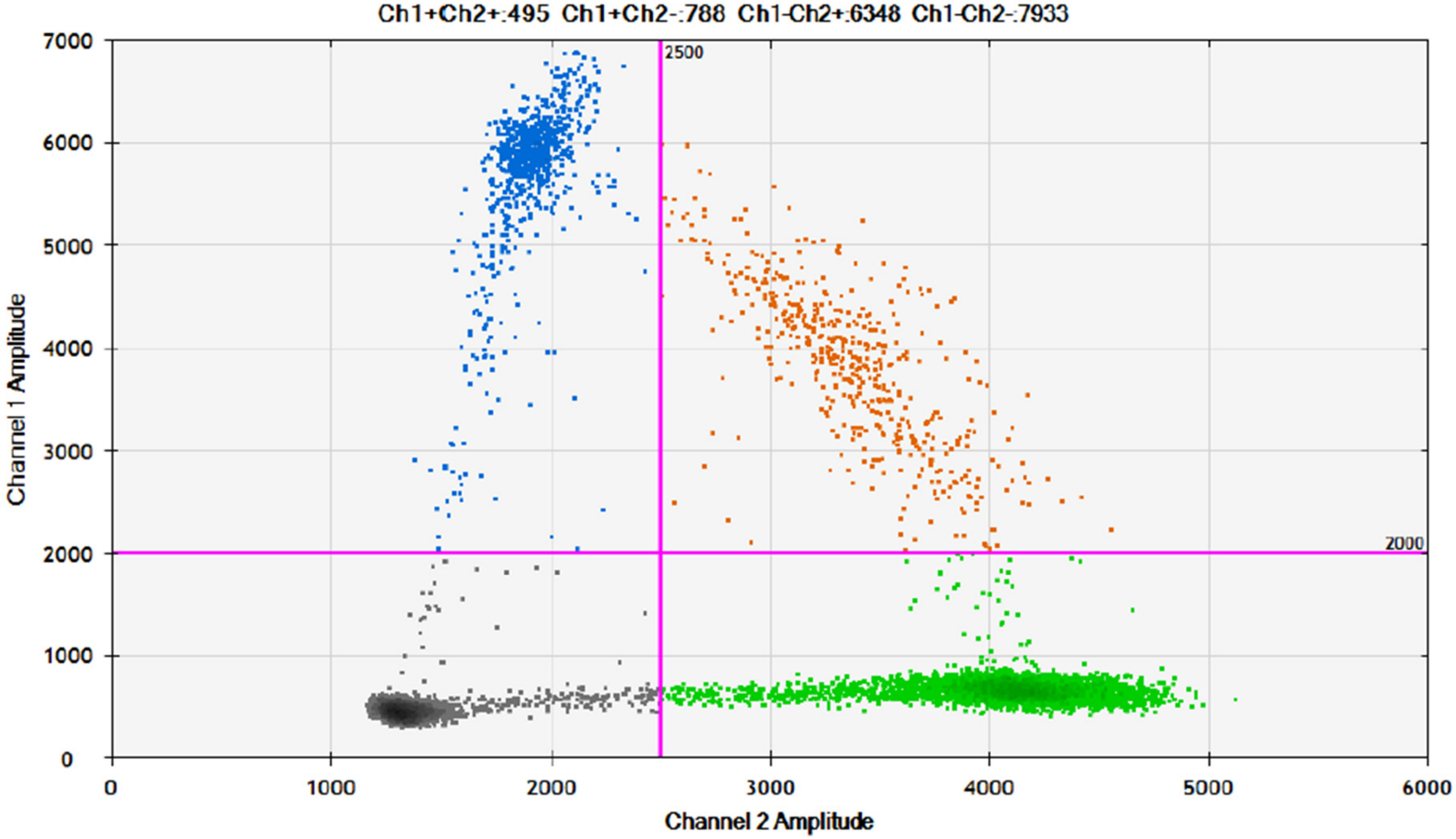

Accepted Droplets	Concentration（copies/ul）	Copies Per 20uL Well	ddPCR VAF	DNA loaded（ng）
15564	101	2020	12.92%	30.00
	681	13620		
