# Supplementary material for: Comprehensive genomic profiling of high‐grade serous ovarian carcinoma from Chinese patients identifies co‐occurring mutations in the Ras/Raf pathway with TP53
Source: Cancer Med. 2019 May 24;8(8):3928–35. doi: 10.1002/cam4.2243 (PMC6639185; doi:10.1002/cam4.2243)

All 19 co-occurring mutations of p53 and RAS/RAF (Sanger validated / ddPCR validated / not validated)

Individual ID	TP53 mutation	VAF (TP53)	KRAS mutation	VAF (KRAS)	NRAS mutation	VAF (NRAS)	BRAF mutation	VAF (BRAF)
Pt9	c.782+1G>A	0.44	p.G12V	0.50				
Pt13	p.R248W	0.06	p.G12D	0.01				
	p.Y205C	0.01						
Pt14	p.M243_G244del	0.43	p.A59G	0.17				
Pt43	P.N239*	0.26	p.G12V	0.42				
Pt69	p.C277F	0.57	p.G12A	0.34				
Pt30	p.P64Afs*85	0.41			p.Q61R	0.42		
Pt51	p.R342*	0.15			p.G12C	0.12		
Pt59	p.R273H	0.87			p.Q61K	0.41		
Pt36	c.920-2A>G	0.67					p.D594N	0.37

Pt9

TP53 NM_000546 c.782+1G>A

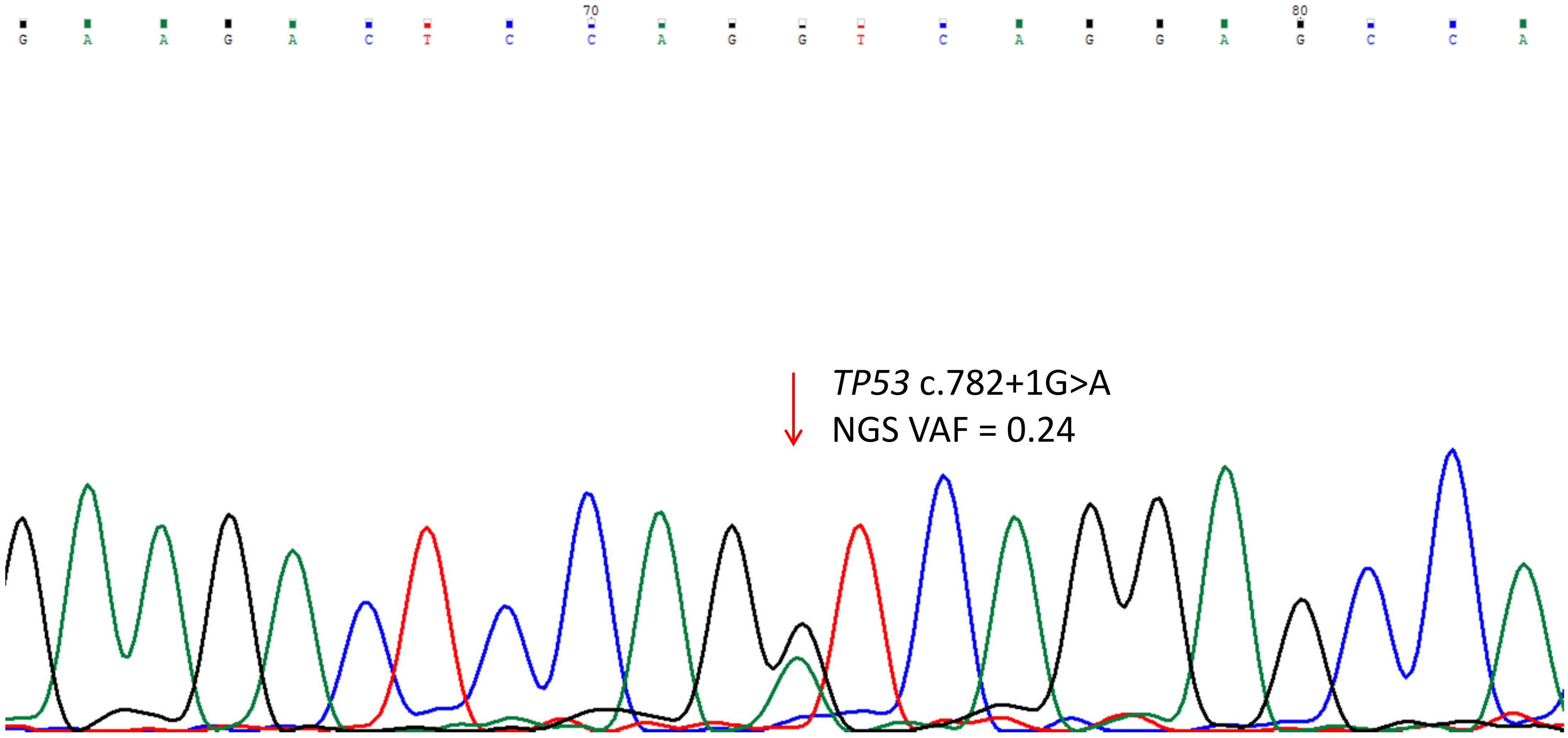

Pt9

KRAS NM_004985 c.35G>T p.G12V

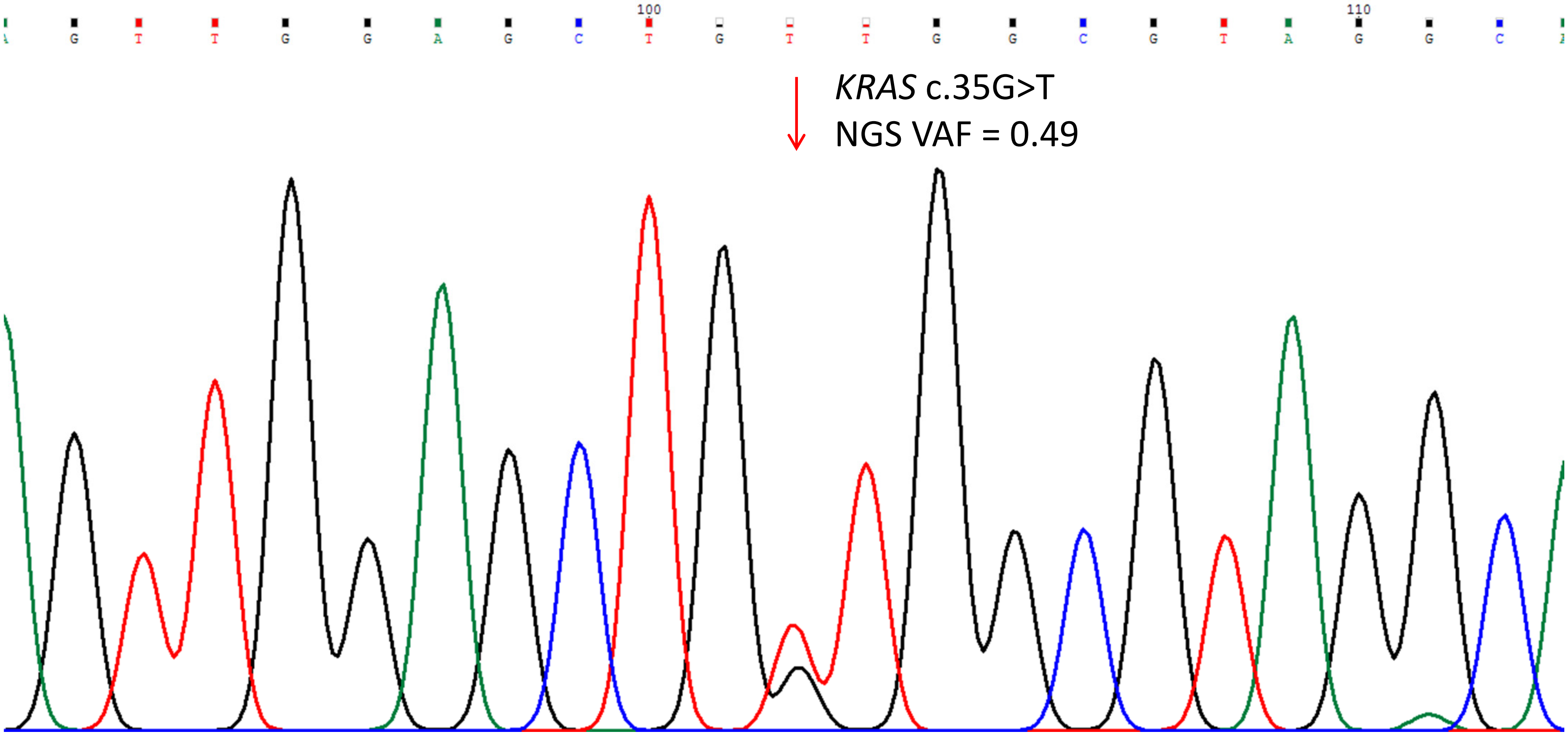

Pt13

TP53 NM_000546 c.742C>T p.R248W

NGS VAF = 0.06

Sanger failed. Validated by ddPCR (VAF = 0.0693)

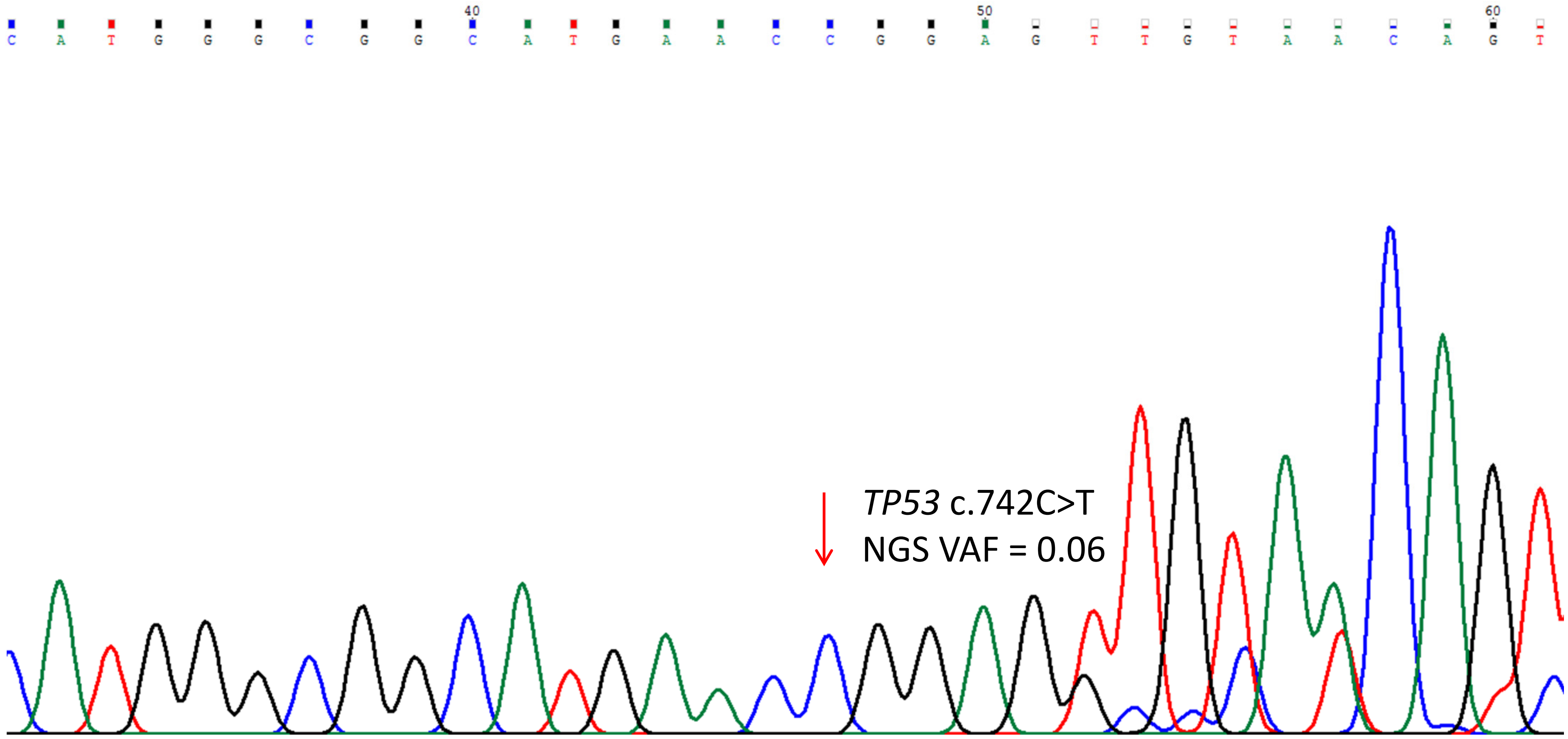

Pt13

TP53 NM_000546 c.614A>G p.Y205C

NGS VAF = 0.01

Sanger failed. No ddPCR primer available.

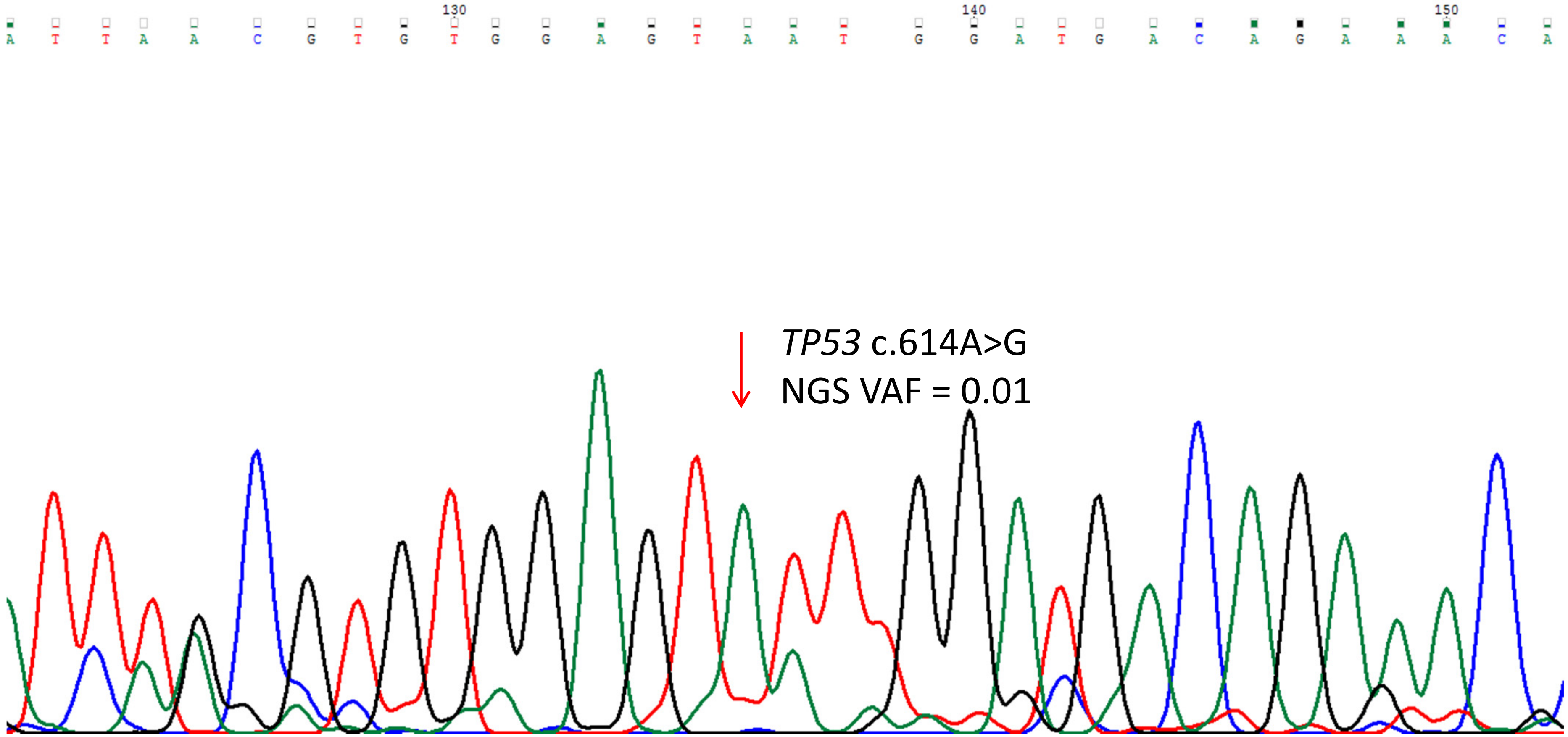

Pt13

KRAS NM_004985 c.35G>A p.G12D

NGS VAF = 0.01

Sanger failed. Validated by ddPCR (VAF = 0.0067)

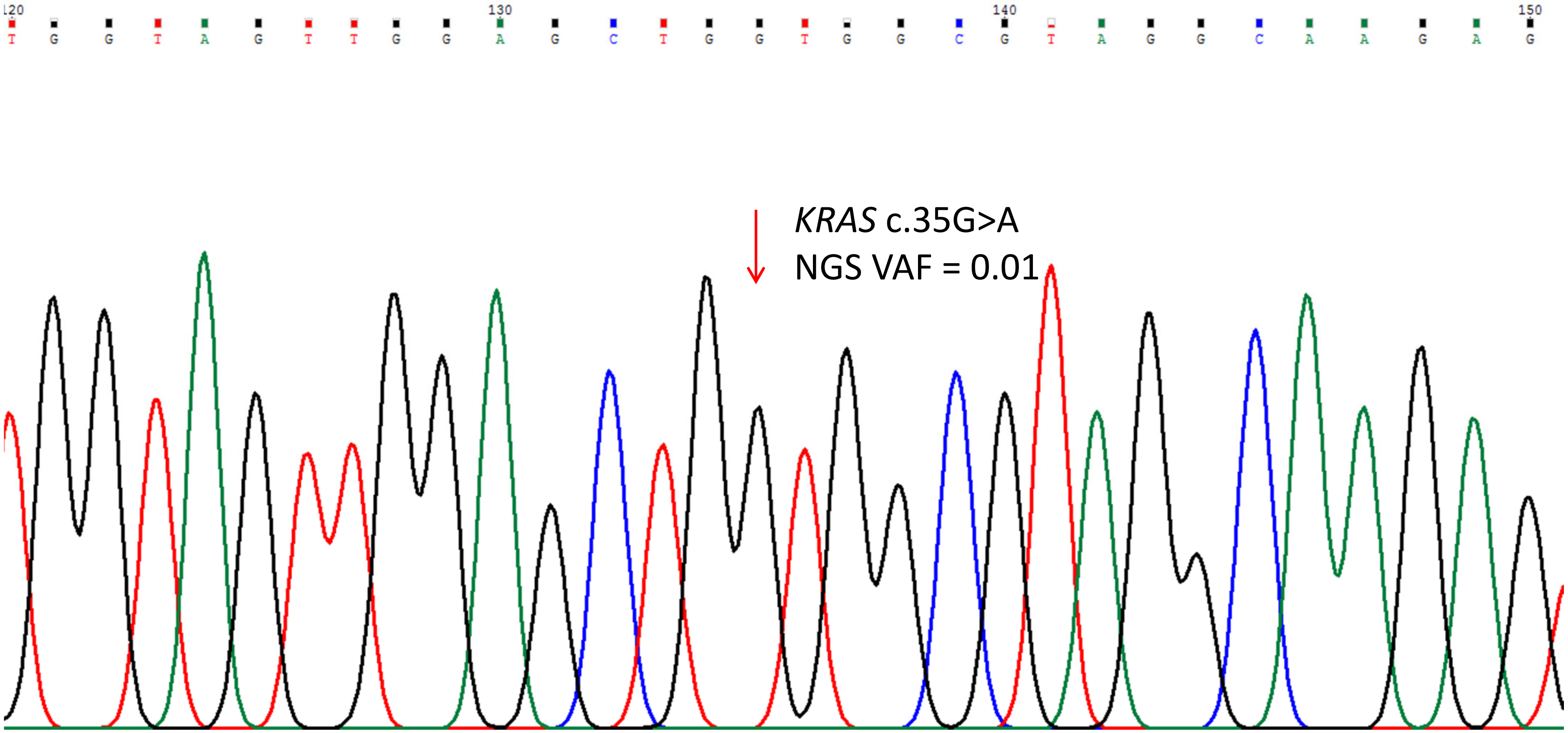

Pt14

TP53 NM_000546 c.727_732del p.M243_G244del

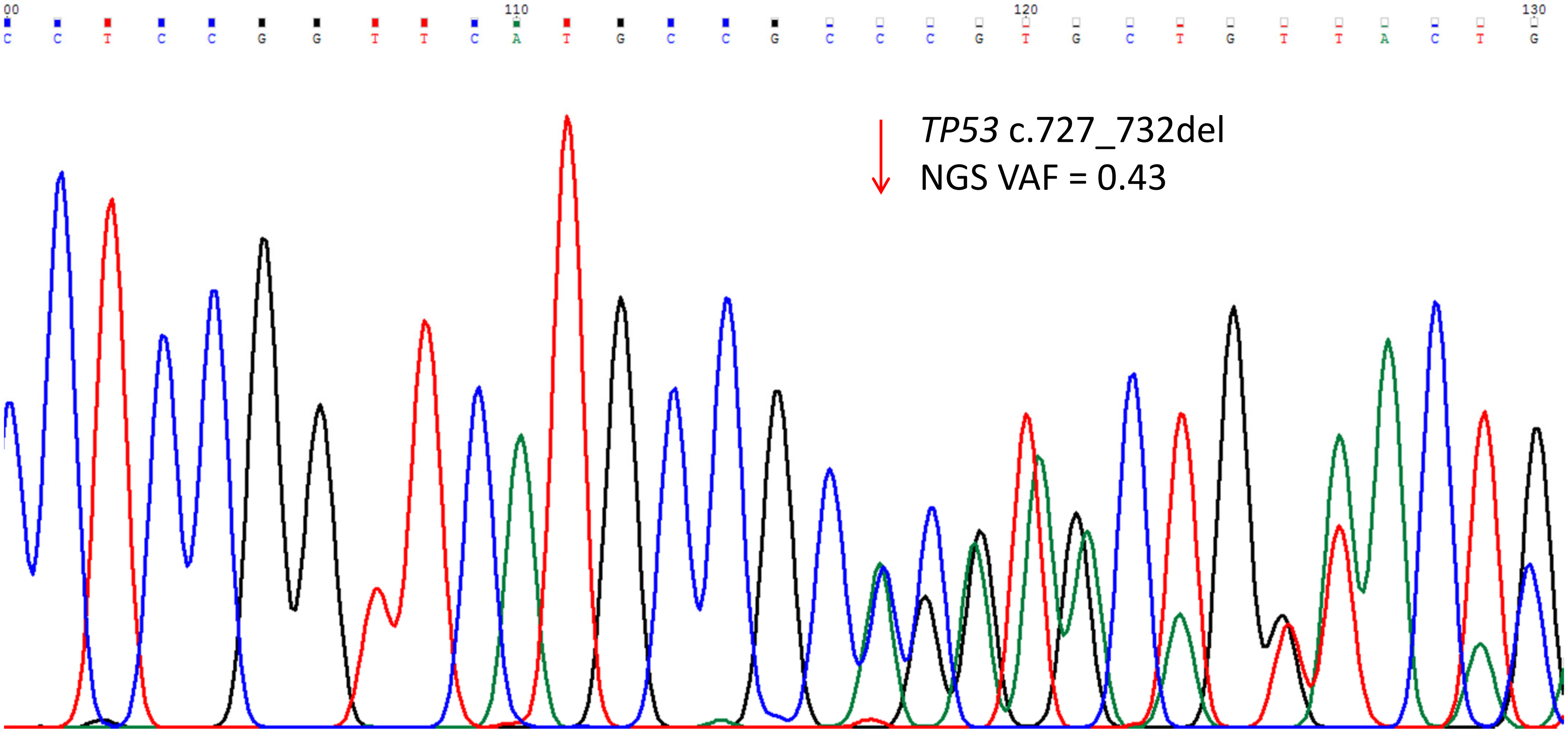

Pt14

KRAS NM_004985 c.176C>G p.A59G

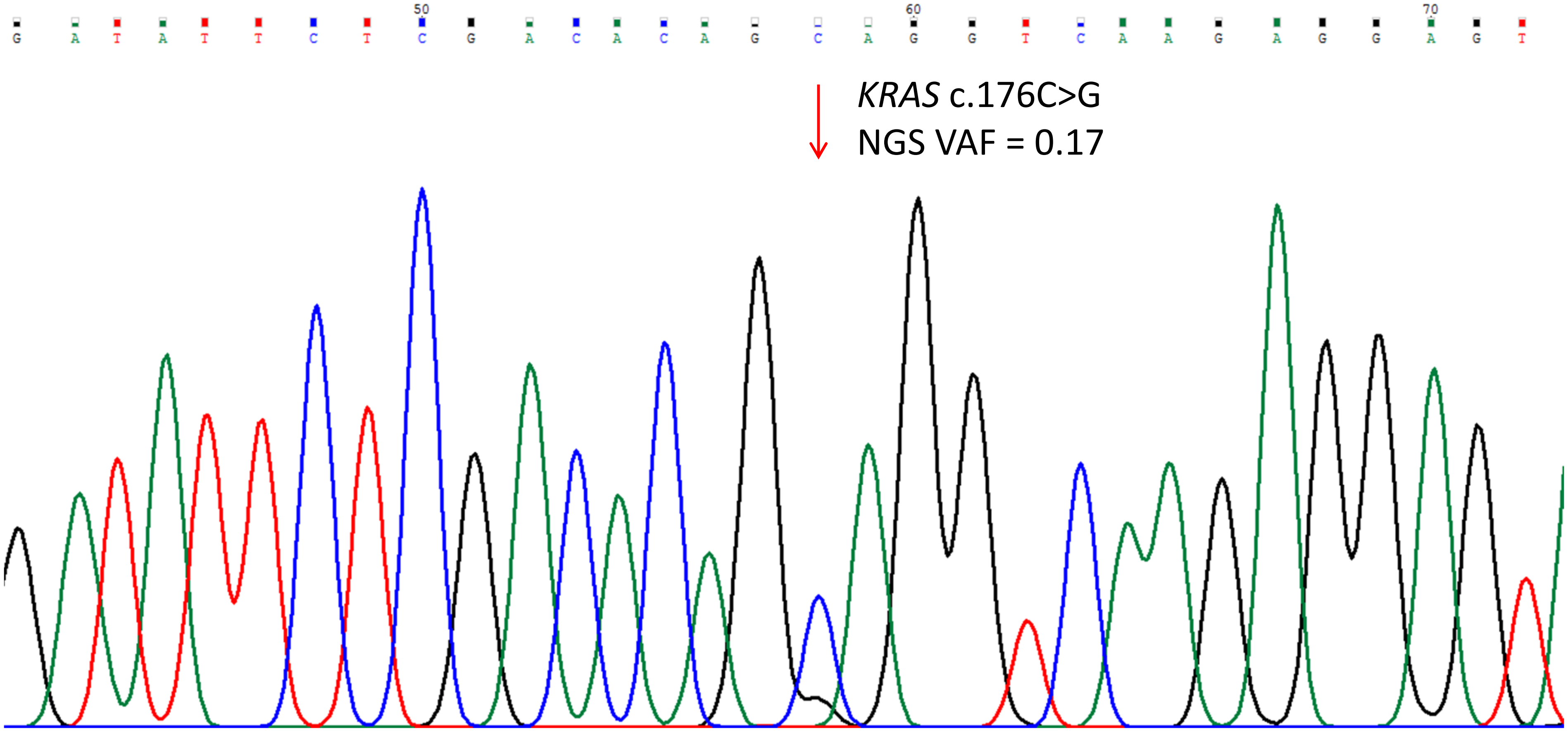

Pt43

TP53 NM_000546 c.714dup P.N239*

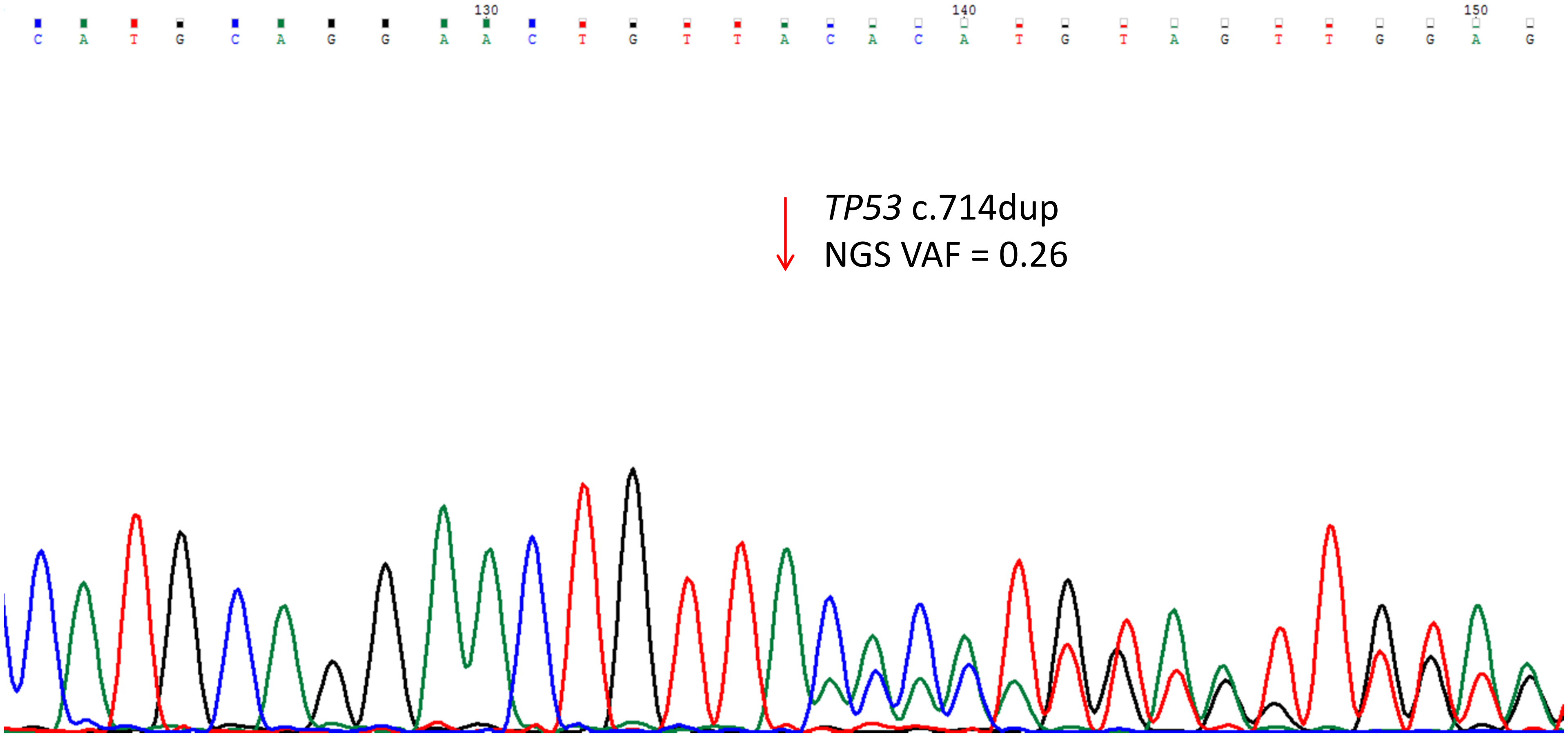

Pt43

KRAS NM_004985 c.35G>T p.G12V

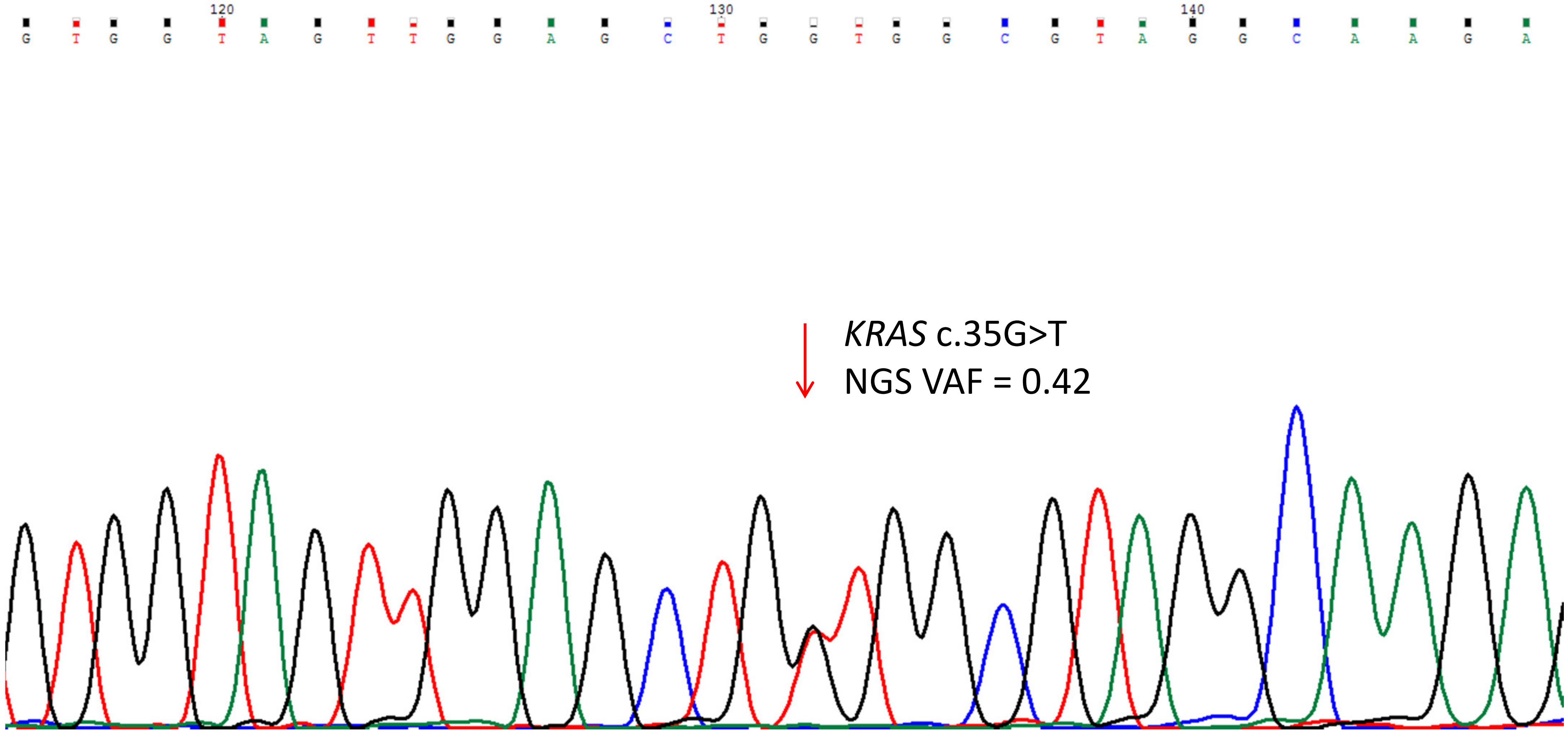

Pt69

TP53 NM_000546 c.830G>T p.C277F

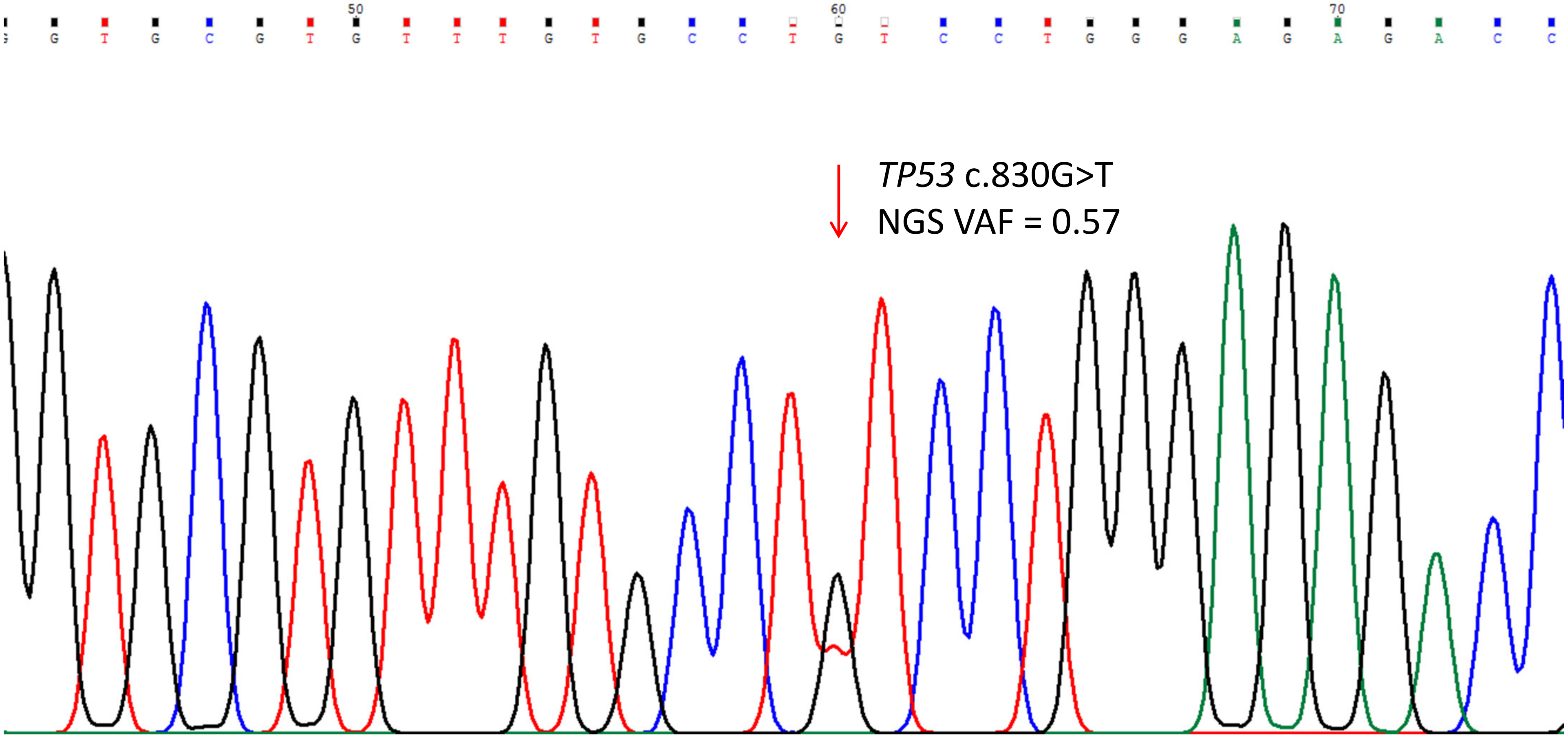

Pt69

KRAS NM_004985 c.35G>C p.G12A

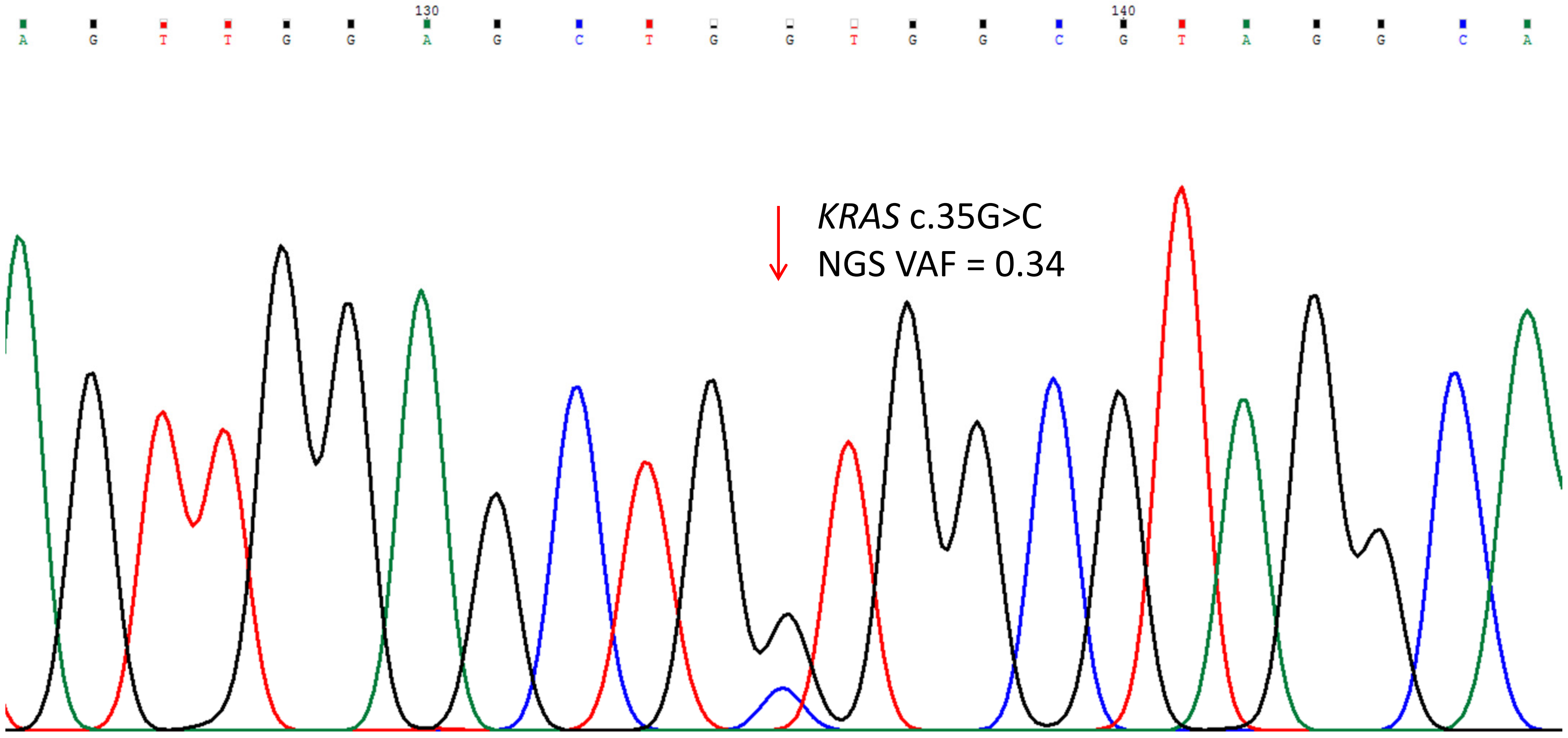

Pt30

NRAS NM_002524 c.182A>G p.Q61R

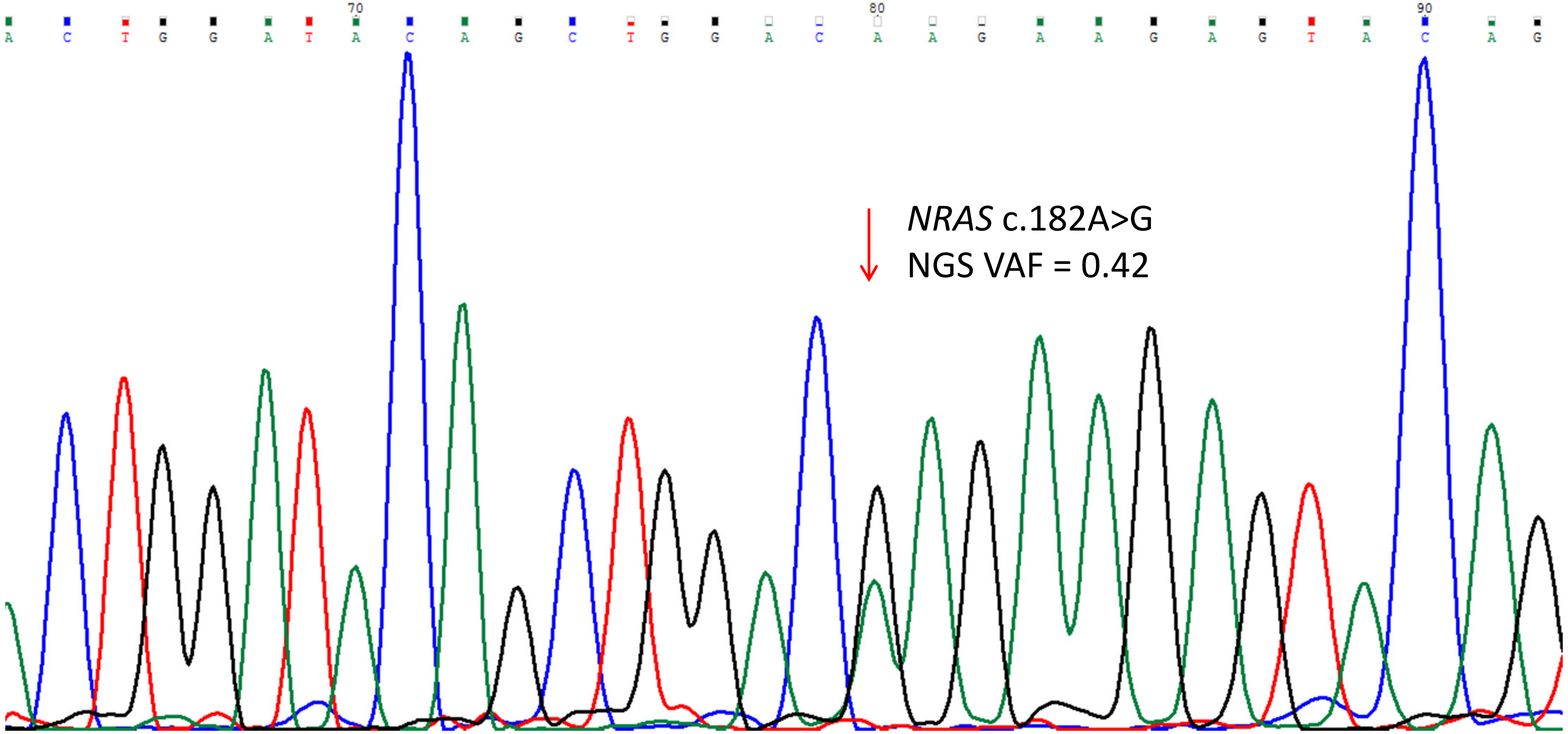

Pt30

TP53 NM_000546 c.189_190insG p.P64Afs*85

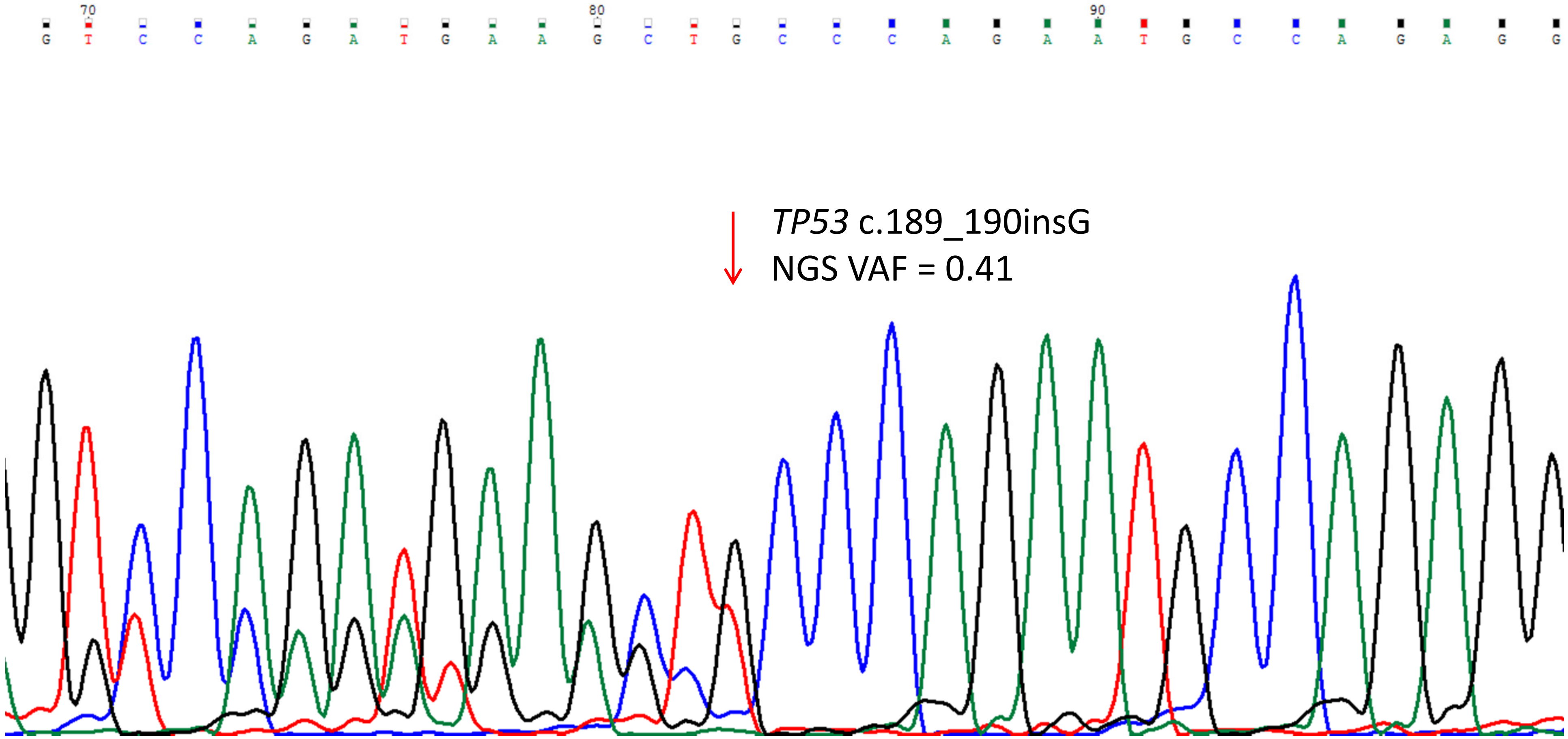

Pt51

TP53 NM_000546 c.1024C>T p.R342*

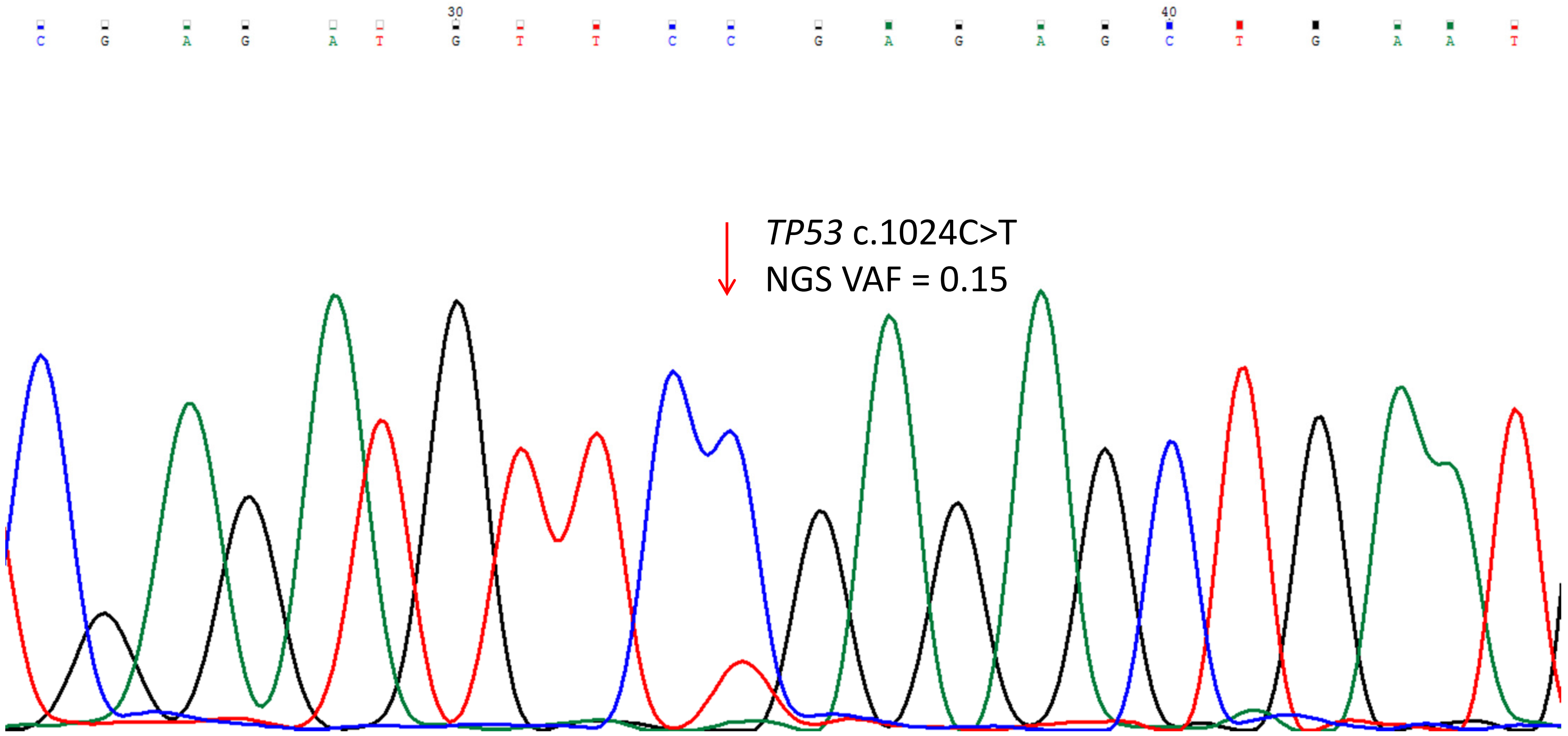

Pt51

NRAS NM_002524 c.34G>T p.G12C NGS VAF = 0.12

Sanger failed. Validated by ddPCR (VAF = 0.1292)

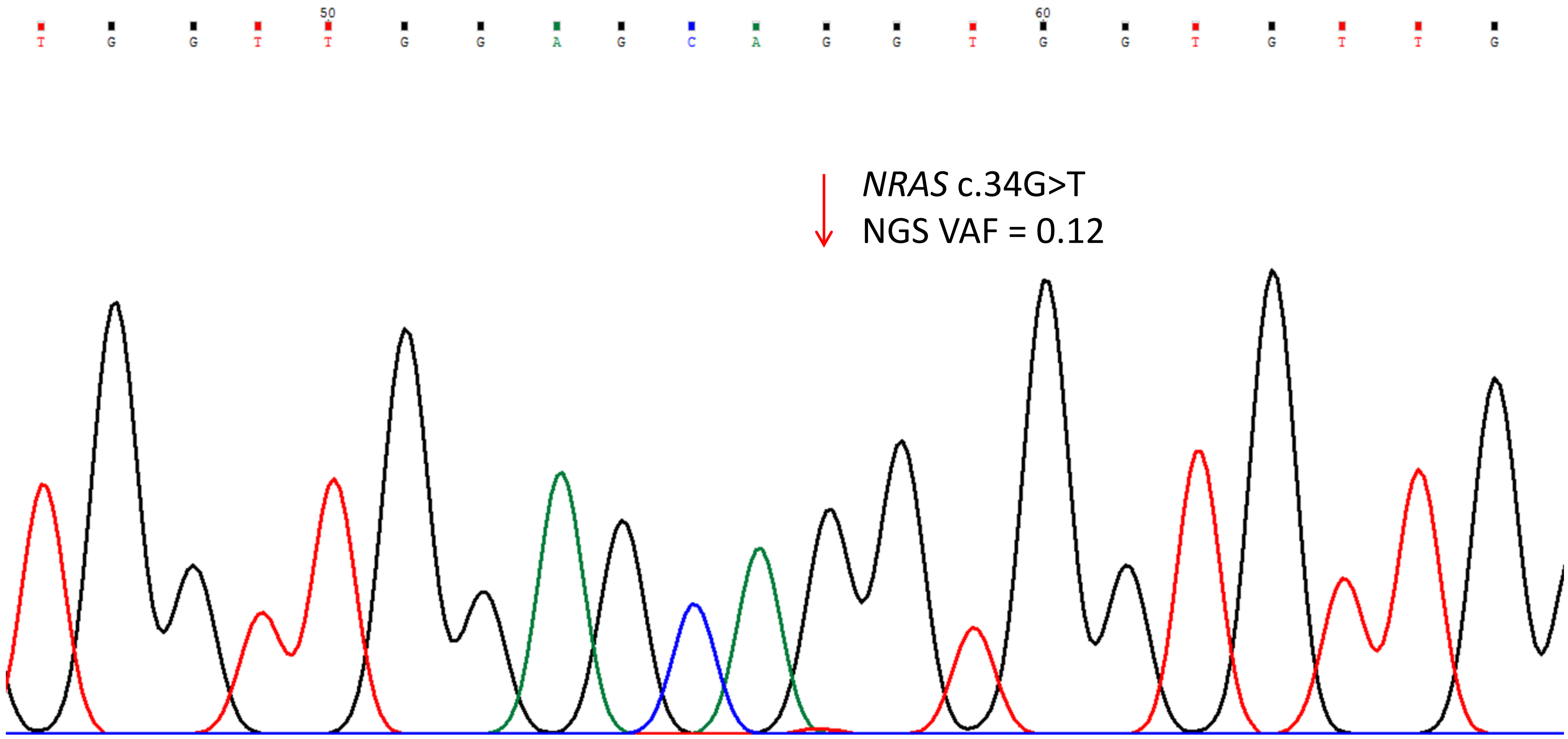

Pt59

TP53 NM_000546 c.818G>A p.R273H

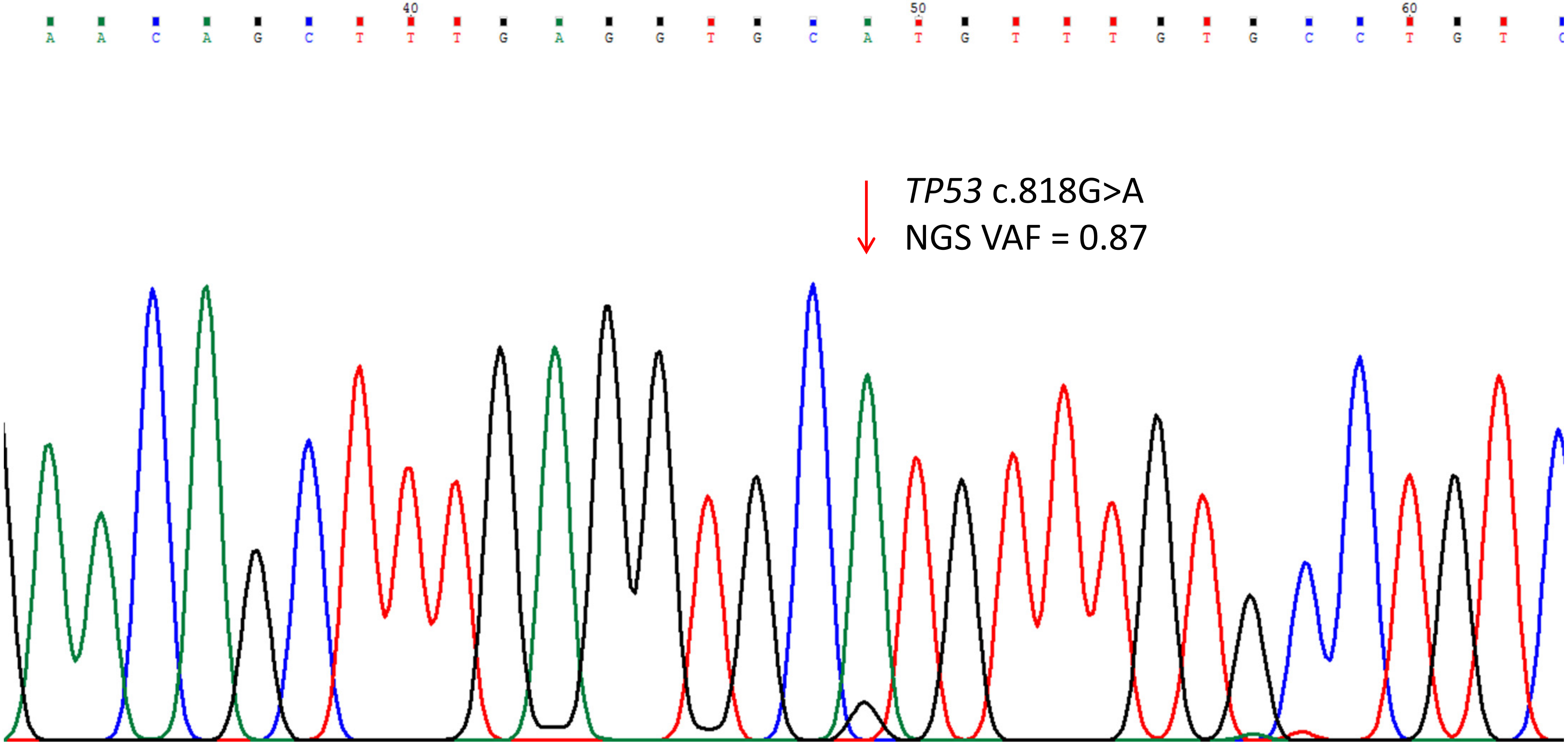

Pt59

NRAS NM_002524 c.181C>A p.Q61K

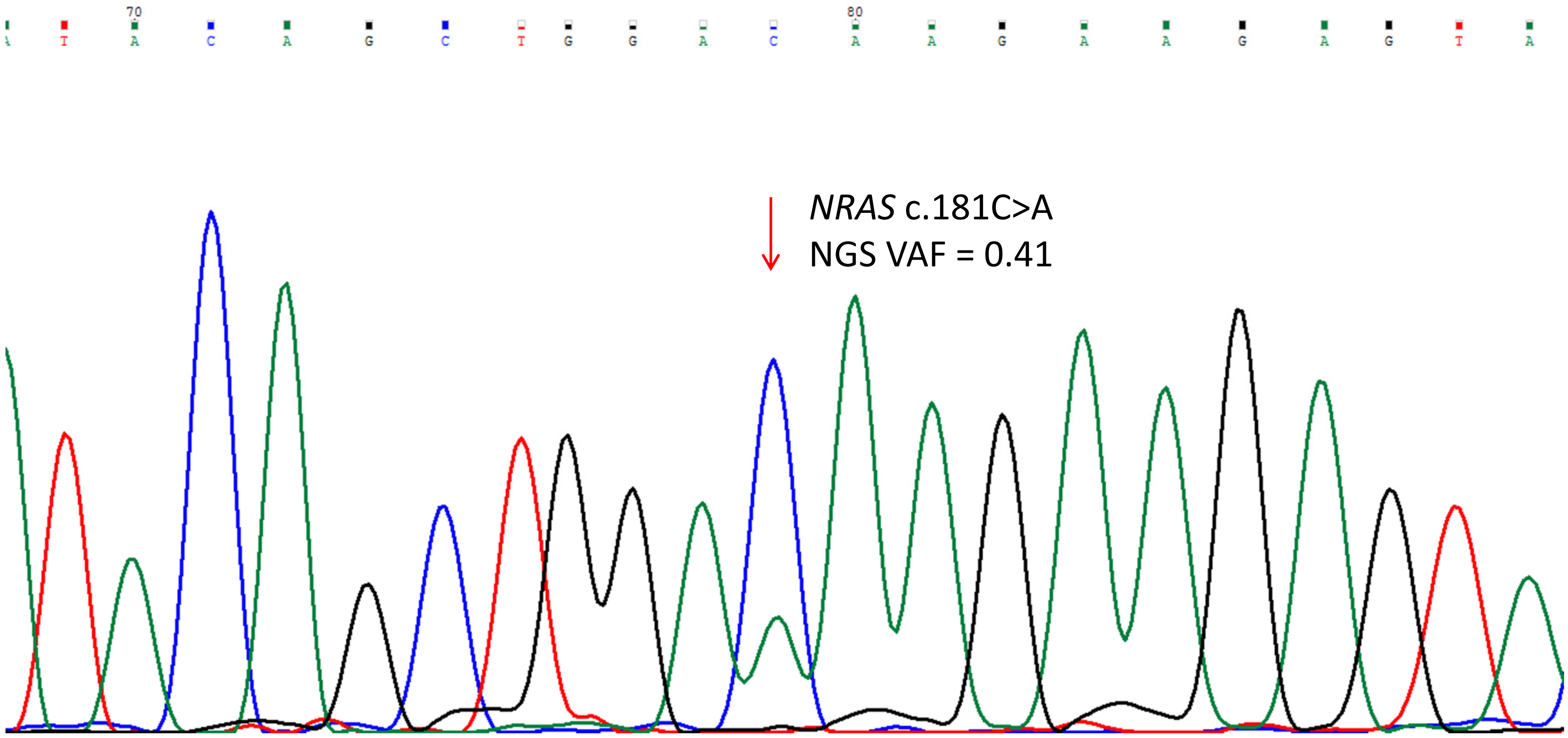

Pt36

TP53 NM_000546 c.920-2A>G

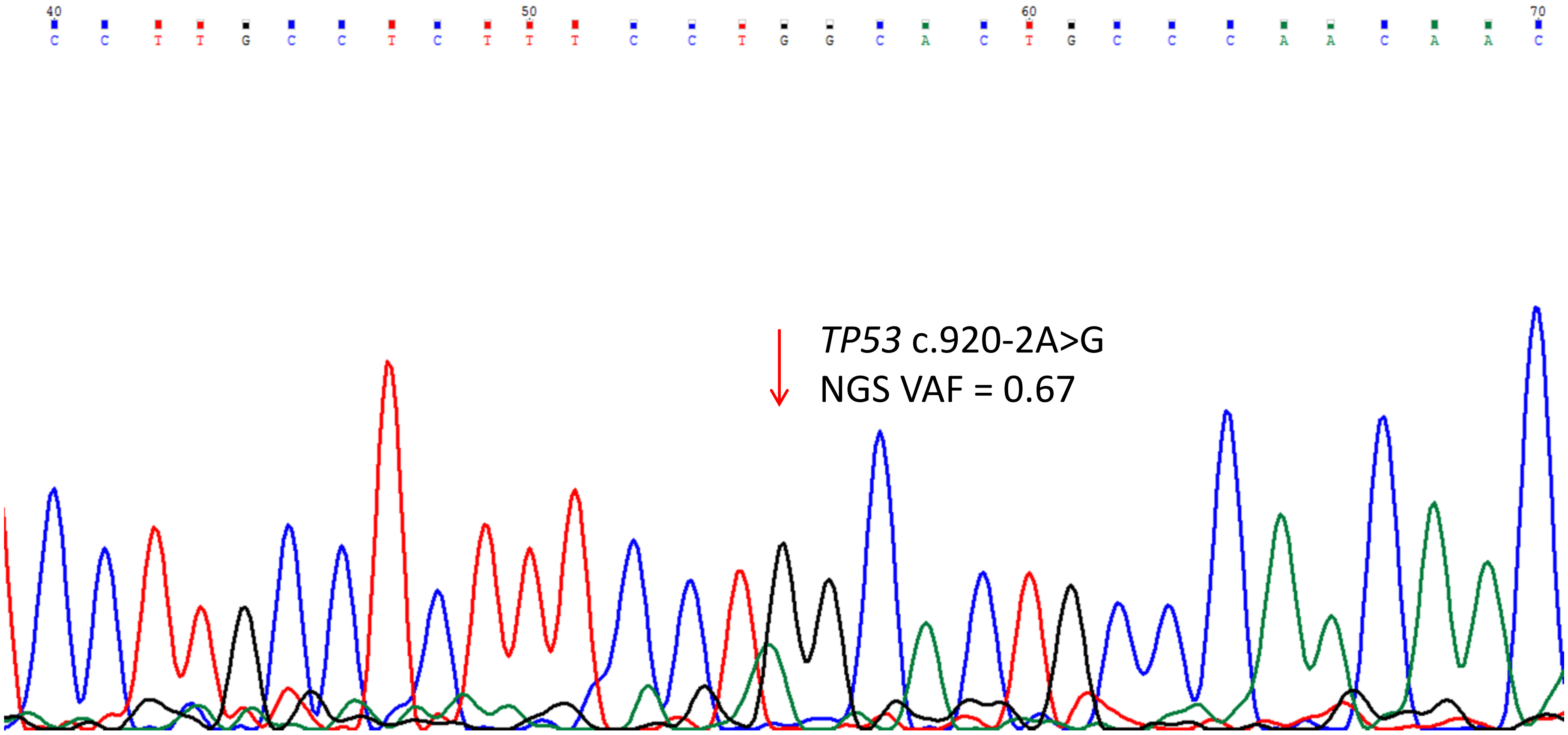

Pt36

BRAF NM_004333 c.1780G>A p.D594N

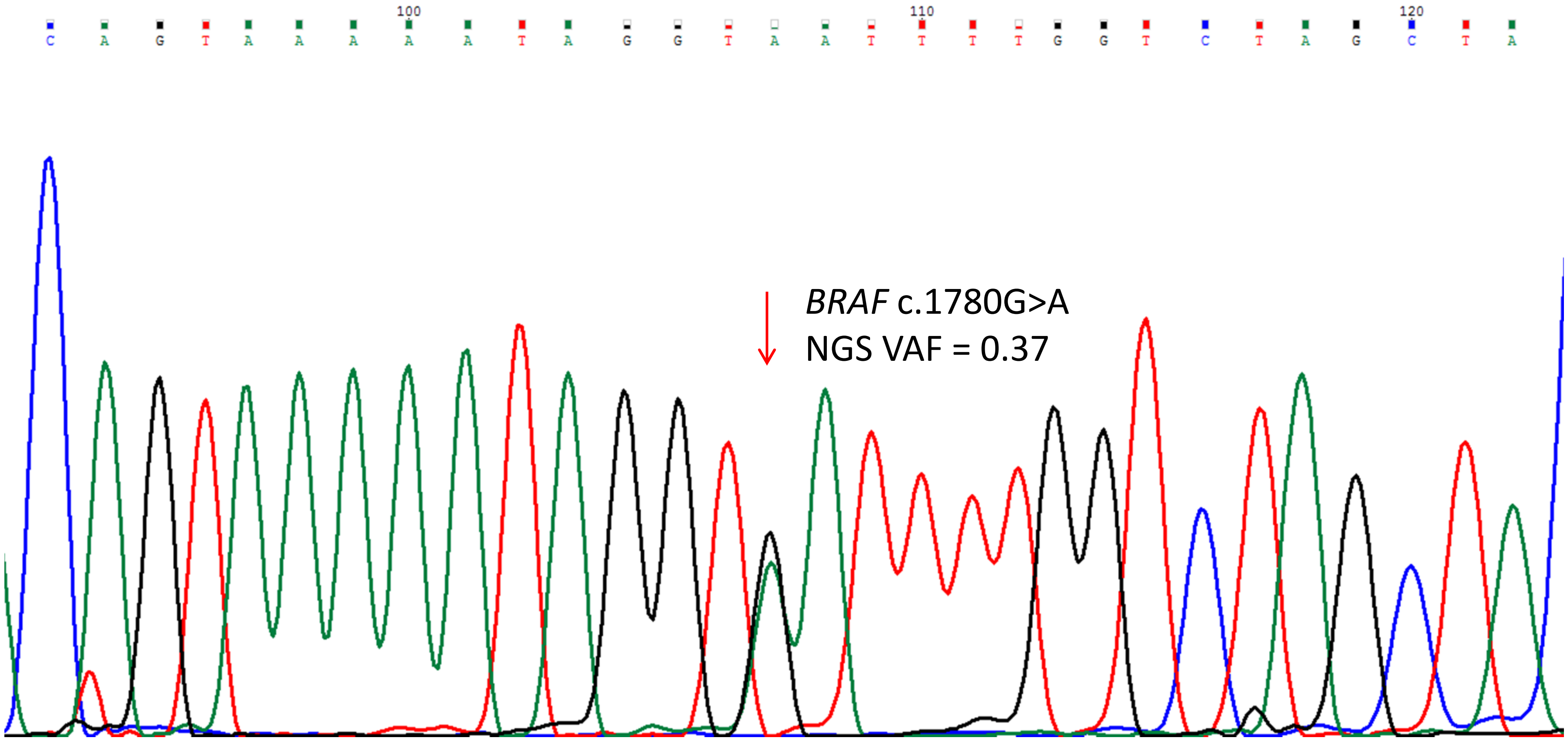

Supplement: Supplementary file 5 [file CAM4-8-3928-s005.pdf]
